# Dual attenuation of proteasomal and autophagic BMAL1 degradation in *Clock*^*Δ19*/+^ mice contributes to improved glucose homeostasis

**DOI:** 10.1038/srep12801

**Published:** 2015-07-31

**Authors:** Kwon Jeong, Baokun He, Kazunari Nohara, Noheon Park, Youngmin Shin, Seonghwa Kim, Kazuhiro Shimomura, Nobuya Koike, Seung-Hee Yoo, Zheng Chen

**Affiliations:** 1 Department of Biochemistry and Molecular Biology, The University of Texas Health Science Center at Houston, 6431 Fannin St., Houston, TX 77030; 2 Department of Neuroscience, The University of Texas Southwestern Medical Center, 5323 Harry Hines Blvd., Dallas, TX 75390; 3 Department of Neurobiology and Physiology, Center for Sleep and Circadian Biology, Northwestern University, 2205 Tech Drive, Evanston, IL 60201; 4 Department of Physiology and Systems Bioscience, Kyoto Prefectural University of Medicine, Kyoto, 602-8566, Japan

## Abstract

Circadian clocks orchestrate essential physiology in response to various cues, yet their mechanistic and functional plasticity remains unclear. Here, we investigated *Clock*^*Δ19*/+^ heterozygous (Clk/+) mice, known to display lengthened periodicity and dampened amplitude, as a model of partially perturbed clocks. Interestingly, Clk/+ mice exhibited improved glycemic control and resistance to circadian period lengthening under high-fat diet (HFD). Furthermore, BMAL1 protein levels in Clk/+ mouse liver were upregulated compared with wild-type (WT) mice under HFD. Pharmacological and molecular studies showed that BMAL1 turnover entailed proteasomal and autophagic activities, and CLOCKΔ19 attenuated both processes. Consistent with an important role of BMAL1 in glycemic control, enhanced activation of insulin signaling was observed in Clk/+ mice relative to WT in HFD. Finally, transcriptome analysis revealed reprogramming of clock-controlled metabolic genes in Clk/+ mice. Our results demonstrate a novel role of autophagy in circadian regulation and reveal an unforeseen plasticity of circadian and metabolic networks.

Virtually all species have evolved a biological timer, called the circadian clock, to orchestrate diverse bodily physiology according to the geophysical daily cycles of Earth rotation. In mammals, the basic component of the clock system is the cell-autonomous molecular oscillator containing interlocked negative feedback loops^1^. The core loop, consisted of positive (transcriptional activators CLOCK/BMAL1 or NPAS2/BMAL1) and negative (transcriptional repressors PERIOD1/2 *and* CRYPTOCHROME1/2) arms, is responsible for generating molecular rhythms, whereas competing nuclear receptors REV-ERBs and RORs form the secondary or stabilization loop to regulate *Bmal1* expression to confer rhythm stability and robustness^1^. Extensive studies have revealed an elaborate regulatory network governing the function of the molecular oscillator and its components, involving both transcriptional and posttranscriptional mechanisms^2^,^3^,^4^. At the organismal level, the peripheral oscillators are also coordinated by the central pacemaker in the hypothalamic suprachiasmatic nuclei (SCN), establishing a complex hierarchical timing system^1^.

Accumulating evidence has unveiled a critical role of the clock as a master regulator of metabolic and physiological fitness^5^,^6^,^7^. The clock drives expression of a wide array of key metabolic genes in a tissue-specific manner^6^,^8^,^9^. In accordance, proteomic and metabolomic studies have also revealed rhythmic accumulation of proteins and metabolites in metabolically active tissues across the circadian cycle^10^,^11^. In functional studies, circadian misalignment as a result of shift work or artificially imposed laboratory settings was found to cause metabolic abnormalities such as glucose intolerance and hyperinsulinemia in human and mice^12^,^13^. Reciprocally, the clock can also be reset by metabolism, as exemplified by the bidirectional regulation of circadian rhythms and NAD+ biosynthesis^5^,^14^. Such in-depth mechanistic understanding strongly supports a convergence of circadian and metabolic cycles^5^,^15^,^16^.

CLOCK/BMAL1 plays a central role in circadian metabolic regulation. Recent ChIP-seq studies have revealed a largely coincident CLOCK and BMAL1 binding throughout mouse liver genome^8^. Furthermore, genetic studies have also revealed overlapping metabolic dysfunctions in *Clock*- and *Bmal1*-deficient mice^6^,^17^. For example, *Clock*^*Δ19*/*Δ19*^ mutant mice, harboring a dominant negative *Clock* mutant allele, have been shown to develop a myriad of metabolic disorders, including obesity, hyperlipidemia, hepatic steatosis, hyperglycemia, hypoinsulinemia, and respiratory uncoupling, especially with age or high-fat dietary challenge^18^,^19^. Similarly, global or tissue-specific disruption of *Bmal1* has been found to dysregulate energy homeostasis, although the specific effects are highly complex, depending on factors such as tissue type, age, and strain background^20^,^21^. More recently, mice with global *Bmal1* knockout were shown to display impaired AKT phosphorylation and circadian insulin response, concomitantly becoming susceptible to obesity when fed with high-fat diet starting at a young age^22^. Although the evidence clearly established a key role of CLOCK/BMAL1 in energy metabolism, much work remains to delineate the underlying mechanisms, especially given the aforementioned confounding factors.

Complete loss of circadian rhythms in human is rare; rather, our clocks mainly suffer from misaligned phase (jet-lag, sleep disorders, shift work) or dampened amplitude (aging or chronic diseases)^1^,^23^. Therefore, studies of partially compromised, rather than completely disrupted, clocks are highly relevant for our well-being^24^. The importance of understanding partially perturbed clocks is also highlighted by recent small molecule studies. Specifically, clock-enhancing molecules (CEMs) were able to potentiate circadian amplitude of reporter rhythms in WT cells with intact clocks or *Clock*^*Δ19*/+^ heterozygous cells where a weaker (1/3 of normal amplitude) circadian clock persists, yet failed to show any effects in clock-disrupted cells, either *Clock*^*Δ19*/*Δ19*^ or *Bmal1*^−/−^23,25. Furthermore, the complex clock regulatory mechanisms at both transcriptional and post-transcriptional levels also permit compensatory adaptation in response to compromising challenges. Quantitative siRNA mediated knockdown of *Clock* revealed dose-dependent changes in expression of its output genes *Period2* as well as compensatory enrichment of both paralogous and non-paralogous clock components (*Npas2* and *Bmal1*, respectively)^26^. At the post-transcriptional level, heterodimerization of CLOCK and BMAL1 has been shown to be required for codependent phosphorylation, which promotes their transactivation and CLOCK nuclear localization and subsequent proteasomal degradation^27^,^28^. Interestingly, the *Clock*^*Δ19*^ mutant form, lacking the transactivation domain and thus missing two key phosphorylation sites, was more stable than the wild-type CLOCK^28^. Thus, partially disrupted clocks may remain capable of adapting within the core oscillator and systemically in the output network to various challenges, consequently rescuable by cognate therapeutic regimens.

To shed more light on partially disrupted clocks and delineate mechanistic and functional characteristics, we employed the *Clock*^*Δ19*/+^ heterozygous mice (Clk/+) previously shown to display a 1-h lengthening of the free-running period and partial dampening of circadian amplitude^29^. Given the demonstrated strong decrease in behavioral and molecular oscillatory amplitude and consequently robust (type 0) phase resetting, these mice have been proposed as a circadian aging model^30^. However, little is known regarding the clock network and physiological output in this mouse mutant. Since *Clock*^*Δ19*/*Δ19*^ homozygous mutant mice (Clk/Clk) suffer from various metabolic disorders under high-fat diet (HFD) conditions^18^, we employed the HFD challenge to examine circadian and metabolic regulation in Clk/+ mice. We observed molecular, metabolic and behavioral adaptations indicative of strong circadian plasticity in the Clk/+ background. Our molecular studies further revealed an underlying mechanism involving dual attenuation of proteasomal and autophagic turnover of BMAL1 by the CLOCKΔ19 mutant.

## Results

### Clk/+ mice displayed improved glucose homeostasis relative to WT under high-fat diet

**Figure 1:**
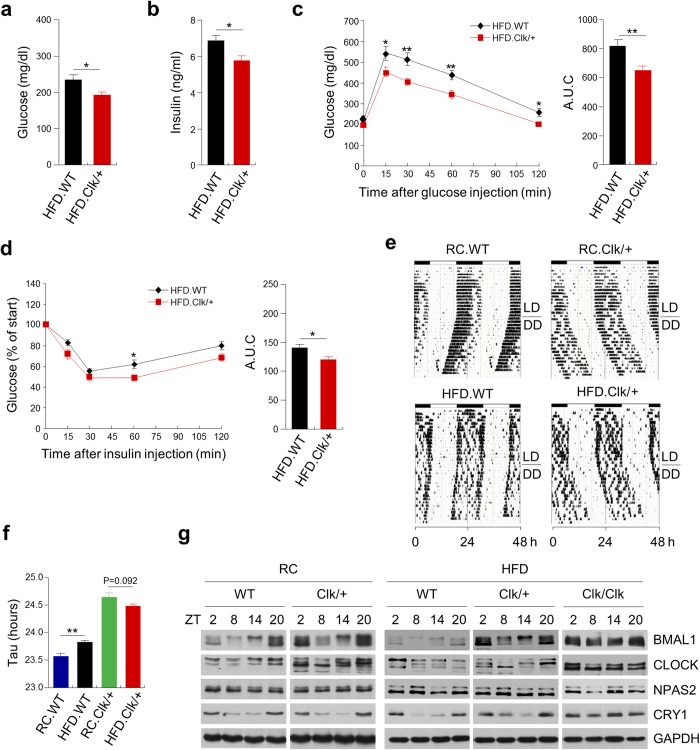
*Clock*^*Δ19*/+^ (Clk/+) mutant mice displayed improved glucose homeostasis and resistance to period lengthening and BMAL1 depletion under high-fat diet (HFD) feeding. Fasting glucose (**a**) serum insulin (**b**) glucose tolerance (**c**) and insulin tolerance (**d**) in WT and Clk/+ mutant mice after HFD feeding for 12 weeks (n = 9 − 11). For (**c**) and (**d**) area under curve (A.U.C) comparison is shown next to the graphs. (**e**) Representative actograms in each condition were shown. The mice were kept on a 12 h:12 h light:dark cycle (represented in the bar above) for 16 days and then released into constant darkness. (**f**) Circadian free-running period (tau) was calculated by using the ActiView software (n = 3 − 5). (**g**) Liver tissues were collected over the circadian time course from mice fed with regular chow (RC) or high-fat diet (HFD). Lysates were prepared and subjected to immunoblotting analysis using antibodies against clock proteins and GAPDH as control. The results were representative of three independent experiments. *p < 0.05, **p < 0.01, one-way ANOVA.

To determine metabolic adjustments in Clk/+ mice with a partially perturbed clock, we investigated wild-type (WT) and Clk/+ mice fed with regular chow (RC) or high-fat diet (HFD) under *ad libitum* conditions. With RC, the WT and Clk/+ mice were essentially indistinguishable in body weight, food intake, fasting glucose levels and glucose tolerance and insulin tolerance (Supplementary Fig. 1), indicating a largely normal energy metabolism in Clk/+ under regular chow diet. Next we subjected WT and Clk/+ littermates to HFD feeding. Interestingly, whereas body weight, food intake and serum lipids remained largely constant between the genotypes (Supplementary Fig. 2), significant improvement in glucose homeostasis, as measured by fasting blood glucose levels, serum insulin levels, glucose tolerance and insulin tolerance, was observed in Clk/+ mice compared with WT (Fig. 1a–d). On the contrary, consistent with previous studies showing that *Clock*^*Δ19*/*Δ19*^ homozygous mutant mice (denoted as Clk/Clk) exhibit propensity for metabolic dysfunctions such as obesity and hyperglycemia^18^,^19^, we also observed metabolic dysfunctions in Clk/Clk (Supplementary Fig. 3).

We next evaluated systemic physiological and behavioral parameters of these mice. First we examined circadian wheel-running behavior (Fig. 1e,f). Consistent with previous studies, compared with RC-fed WT, HFD feeding lengthened WT circadian period by approximately 0.3 h (p < 0.05), and RC-fed Clk/+ mice displayed ~1.2 h period lengthening (p < 0.001). Interestingly, period-lengthening by HFD feeding over RC was not significant in Clk/+ mice (RC.Clk/+ 24.6 vs HFD.Clk/+ 24.5 h, p = 0.092), suggesting resistance conferred by *Clock*^*Δ19*^ heterozygosity to HFD-induced circadian period lengthening. Next, we measured systemic energy metabolism using CLAMS metabolic chambers (Supplementary Fig. 4). With HFD feeding, oxygen consumption and heat production were largely unchanged between WT and Clk/+ mice. On the other hand, respiratory exchange ratio (RER) was slightly reduced in Clk/+ relative to WT (Supplementary Fig. 4e,f), suggesting greater utilization of lipid as energy source.

Given the improved glucose homeostasis in Clk/+ in the HFD-induced obesity model, we next investigated a possible effect of *Clock*^*Δ19*^ heterozygosity in *db*/*db* mice (denoted as db), an established mouse genetic model for obesity and diabetes that lacks functional leptin receptors. Interestingly, consistent with the above results from HFD.Clk/+, improved glycemic control was observed in *db*/*db* mice with heterozygous *Clock*^*Δ19*/+^ alleles (denoted as db:Clk/+) relative to *db*/*db* littermates wild-type for *Clock* (db:WT). Both fasting glucose and insulin levels were reduced in db:Clk/+ mice, concordant with greater glucose and insulin tolerance relative to the db:WT mice (Supplementary Fig. 5).

These observations from both diet-induced and genetic mouse models collectively highlighted an intriguing improvement in glycemic control associated with *Clock*^*Δ19*^ heterozygosity, a novel finding that cannot be otherwise inferred from the broad metabolic dysfunctions in *Clock*^*Δ19*/*Δ19*^ homozygous mice.

### Hepatic BMAL1 accumulation was restored in Clk/+ mice under HFD

We next aimed to delineate the concordant changes in the molecular oscillator. In Western blot analysis of mouse liver samples, we found that BMAL1 protein levels were elevated in Clk/+ compared with WT under RC feeding, and remained highly oscillatory (Fig. 1g, left). In HFD, whereas WT mice showed reduced levels and dampened amplitude of BMAL1 than in RC, Clk/+ mice showed equivalent BMAL1 accumulation when compared with RC-fed Clk/+ (Fig. 1g, right), suggesting the HFD-induced reduction in hepatic BMAL1 seen in WT was restored in Clk/+. This effect was BMAL1 specific as several other core clock component examined did not show this diet-mutation interaction (Fig. 1g and Supplementary Fig. 6). In Clk/Clk mice with a severely disrupted clock, oscillation of several clock components including BMAL1 was largely abolished (Fig. 1g, right and Supplementary Fig. 6).

We further examined clock gene expression in the livers of WT and Clk/+ mice by real-time qPCR analysis (Supplementary Fig. 7). Under RC conditions, oscillatory amplitude of several clock genes was unchanged (*Bmal1*, *Cry1*) or even enhanced (*Npas2*, *Nr1d2, Rorc*) in Clk/+ relative to WT. On the other hand, expression of *Cry2* and *Dbp* was significantly dampened in RC.Clk/+. Consistent with previous results^31^, HFD feeding in WT generally dampened expression of clock genes compared with RC feeding (except *Cry1*, *Per1* and *Rorc*). Such HFD-mediated suppression was retained in Clk/+ for *Per1*, *Npas2*, *Nr1d1* and *Nr1d2*. Interestingly, expression of several clock genes including *Bmal1*, *Npas2, Rorc* and *Cry2* was elevated in HFD.Clk/+ compared with HFD.WT mice.

To further examine the effect of *Clock*^*Δ19*^ allele on BMAL1 in HFD at a cellular level, we treated WT, Clk/+ and Clk/Clk MEF cells with Dexamethasone (DEX) with or without the C16:0 fatty acid palmitate and monitored BMAL1 protein oscillation. In WT MEF cells, palmitate markedly reduced BMAL1 protein levels during the circadian cycle (Supplementary Fig. 8a), recapitulating the HFD effects on BMAL1 in mouse liver (Fig. 1g). BMAL1 levels in Clk/+ and Clk/Clk MEF cells were augmented relative to WT, and importantly were comparable with or without palmitate treatment throughout the circadian time course. In comparison, levels of CRY1 were slightly reduced in Clk/+ and Clk/Clk mutant cells compared with WT, and palmitate did not significantly affect CRY1 levels in either WT or mutant cells. At the transcript level (Supplementary Fig. 8b), whereas WT MEF cells showed a *Bmal1* expression peak at 12 h which was suppressed by palmitate, the Clk/+ and Clk/Clk MEF cells generally exhibited a lower *Bmal1* mRNA levels devoid of a clear peak, contrary to the increases of BMAL1 protein levels in Clk/+ and Clk/Clk MEF cells compared with WT MEF cells.

Taken together, the above liver and MEF cell studies indicated a *Clock*^*Δ19*^-dependent compensatory mechanism to counteract a specific effect of HFD and palmitate to reduce BMAL1 abundance, likely via a post-transcriptional mechanism.

### CLOCKΔ19 proteins stabilized BMAL1

**Figure 2:**
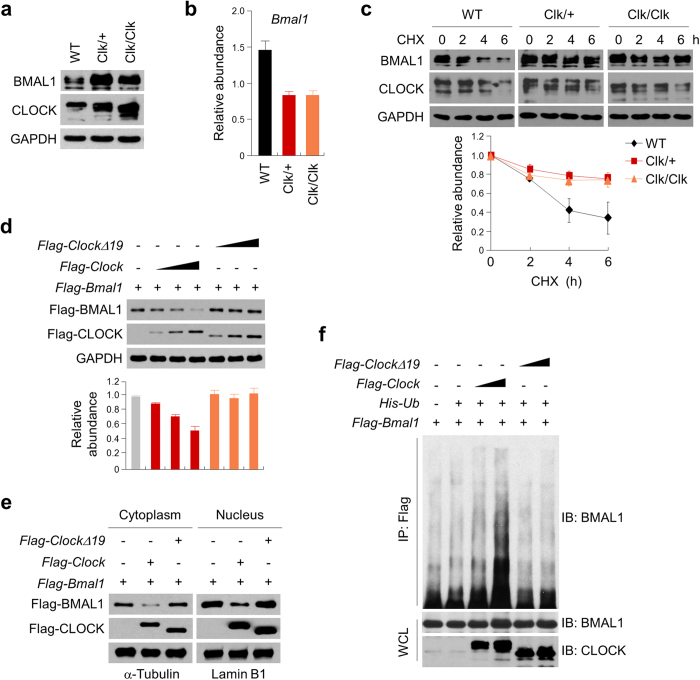
CLOCK and CLOCKΔ19 exerted differential effects on BMAL1 ubiquitination and stability. (**a**,**b**) Protein lysates and total RNAs were prepared from wild-type (WT), *Clock*^*Δ19*/+^ (Clk/+) and *Clock*^*Δ19*/*Δ19*^ (Clk/Clk) MEF cells and subjected to Western blot analysis of CLOCK and BMAL1 (**a**) and real-time qPCR analysis of *Bmal1* transcript levels (**b**). GAPDH was used as a loading control in (**a**). (**c**) WT, Clk/+ and Clk/Clk MEF cells were exposed to cycloheximide (CHX; 25 μg/ml) for the indicated times, and BMAL1 levels were assessed by immunoblotting analysis. Quantification is presented in the lower panel. Error bars represent mean ± SD (n = 3). Half-life was determined by using the GraphPad Prism software with nonlinear, one-phase exponential decay analysis. The analysis calculated the half-life parameter K at 0.191/h, 0.050/h, and 0.054/h in WT, Clk/+ and Clk/Clk MEF cells, corresponding to half-life at 3.63 h, 14.0 h and 12.9 h respectively. The K values are significantly different among all three conditions: p < 0.0001. Half-life was determined by using nonlinear, one-phase exponential decay analysis. Half-life parameter, K, is significantly different in all three conditions: p < 0.0001. (**d**) 293T cells transfected with expression vectors for Flag-BMAL1, Flag-CLOCK and/or Flag-CLOCKΔ19 were subjected to immunoblotting analysis with antibodies against the Flag-tag and GAPDH (loading control). Quantitation of three independent experiments is presented in the lower panel. (**e**) Cytoplasmic and nuclear fractions of 293T cells transfected with the indicated expression vectors were subjected to immunoblotting analysis with antibodies against anti-Flag, α-Tubulin and Lamin B1. The data represent the mean ± SD of three independent experiments. (**f**) 293T cells transfected with the indicated expression vectors for Flag-BMAL1, His-tagged ubiquitin (*His-Ub*), Flag-CLOCK, and Flag-CLOCKΔ19 were lysed and immunoprecipitated with anti-Flag antibody prior to immunoblotting analysis with anti-BMAL1 and -CLOCK antibodies. The relative levels of the targeted proteins were measured by densitometry using the Image J program and the ratios were calculated relative to the GAPDH control.

CLOCK plays an essential role in ubiquitin-dependent BMAL1 degradation concomitant with transactivation by the CLOCK/BMAL1 heterodimer of target promoters^28^,^32^. Many unstable transcription factors are known to activate target gene expression coupled with their own degradation through the ubiquitin-proteasome system^33^. To determine whether CLOCK and the transactivation-deficient CLOCKΔ19 differentially affect BMAL1 degradation, we assessed the endogenous BMAL1 stability in WT, Clk/+, and Clk/Clk MEF cells. BMAL1 protein levels were strongly enhanced, whereas *Bmal1* transcript levels significantly reduced in the Clk/+ and Clk/Clk MEF cells over WT (Fig. 2a,b), supportive of a role of post-transcriptional BMAL1 regulation. We next measured endogenous BMAL1 half-life by treating MEF cells with cycloheximide (CHX) (Fig. 2c). Endogenous BMAL1 levels in WT cells rapidly diminished, with an estimated half-life of 3.6 h. In Clk/+ and Clk/Clk cells, BMAL1 half-life was extended to 13.9 h and 12.9 h respectively, indicating a BMAL1-stabilizing effect of CLOCKΔ19. This effect appeared specific for BMAL1 because CRY1 half-life did not show significant interaction with the mutant allele (Supplementary Fig. 9).

To gain further insight into the role of the CLOCK proteins in BMAL1 regulation, we introduced ectopic expression of Flag-tagged BMAL1 (*Flag-Bmal1*), Flag-tagged CLOCK (*Flag-Clock*) or CLOCKΔ19 (*Flag-ClockΔ19*) to 293T cells. Whereas ectopic expression of Flag-CLOCK reduced the steady-state abundance of Flag-BMAL1 in a dose-dependent manner, Flag-CLOCKΔ19 expression did not destabilize Flag-BMAL1 (Fig. 2d). We next explored the subcellular mechanism underlying CLOCK-dependent degradation of BMAL1 by subcellular fractionation of 293T cells transfected with *Flag-Bmal1*, *Flag-Clock*, and/or *Flag-ClockΔ19* (Fig. 2e). Interestingly, expression of Flag-CLOCK, but not Flag-CLOCKΔ19, strongly attenuated the amount of ectopically expressed BMAL1 in both the cytoplasm and the nucleus. BMAL1 has been shown to be subjected to ubiquitination-dependent proteasomal degradation^34^,^35^. To differentiate the roles of CLOCK versus CLOCKΔ19 in BMAL1 ubiquitination, 293T cells were co-transfected with His-ubiquitin expression vector (*His-Ub*), *Flag-Bmal1* with *Flag-Clock* or *Flag-ClockΔ19.* Whereas robust BMAL1 polyubiquitination was observed with expression of Flag-CLOCK (Fig. 2f), Flag-CLOCKΔ19 led to much attenuated BMAL1 polyubiquitination. Previously sumoylation of BMAL1 at K259 has been shown to be a priming event for its ubiquitination^34^,^35^. We thus generated an expression construct for Flag-BMAL1 K259R mutant proteins (Supplementary Fig. 10). In 293T cells, Flag-BMAL1 K259R showed increased expression levels over Flag-BMAL1 in the presence of Flag-CLOCK expression (Supplementary Fig. 10). Moreover, in cells transfected with *Flag-Clock*, Flag-BMAL1 K259R indeed displayed significantly lengthened half-life compared with Flag-BMAL1 (Supplementary Fig. 10b, 6.7 vs. 3.4 h). These results thus strongly indicated differential roles of CLOCK and CLOCKΔ19 on BMAL1 K259-dependent ubiquitination and subsequent turnover.

### CLOCKΔ19 attenuated BMAL1 degradation through both proteasomal and autophagic pathways

**Figure 3:**
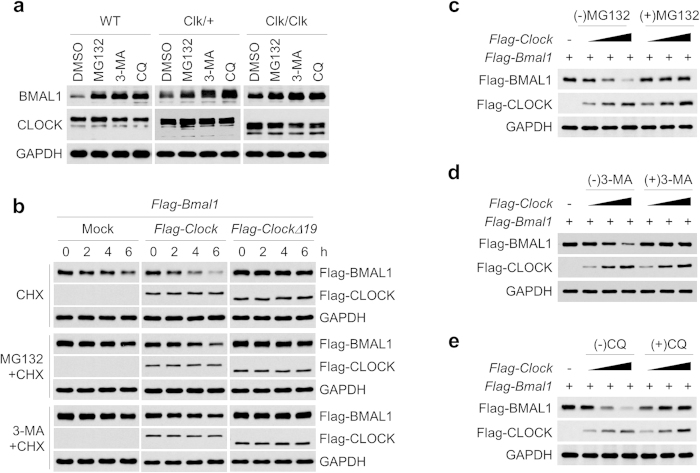
CLOCKΔ19 attenuated BMAL1 degradation via both proteasomal and autophagic pathways. (**a**) WT, Clk/+, and Clk/Clk MEF cells were exposed to 10 μM MG132, 5 mM 3-methyladenine (3-MA) or 25 μM chloroquine (CQ) for 4, 24 and 4 h, respectively. Cell lysates were subjected to immunoblotting analysis with antibodies against BMAL1, CLOCK and GAPDH (loading control). Representative results from three independent experiments are shown. (**b**) 293T cells transfected with the indicated expression vectors were treated with MG132 and 3-MA for 4 and 24 h followed by CHX exposure for the indicated times, and subjected to immunoblotting with antibodies against Flag and GAPDH (loading control). (**c**–**e**) 293T cells transfected with the indicated expression vectors were incubated in the absence or presence of MG132 (10 μM) (**c**), 3-MA (1 mM) (**d**) or CQ (25 μM) (**e**) for 4, 24, and 24 h, respectively. Lysates were subjected to immunoblotting analysis with antibodies against Flag and GAPDH (loading control).

Proteasomal degradation and lysosomal autophagy are two major pathways for cellular turnover of ubiquitinated proteins. Previously, it has been shown that CLOCK and BMAL1 destabilize each other via ubiquitin-dependent proteasomal degradation following heterodimerization and transcriptional activation^32^. On the other hand, despite evidence of clock control of autophagy^36^,^37^, a role of autophagy in clock protein homeostasis has not been reported. To delineate the roles of proteasomal degradation and/or autophagy in BMAL1 turnover, we treated WT, Clk/+ and Clk/Clk MEF cells with MG132 (proteasome inhibitor), 3-MA (3-methyladenine, autophagosome inhibitor) or chloroquine (CQ, lysosome inhibitor). In accordance with previous reports^32^, MG132 exposure attenuated BMAL1 turnover despite different baseline levels in the three MEF cells (Fig. 3a). Interestingly, 3-MA or CQ treatment significantly enriched BMAL1 protein in MEFs to comparable degrees with MG132, indicating that autophagy plays an important role in BMAL1 degradation. On the other hand, ectopic expression of Flag-BMAL1, Flag-CLOCK or Flag-CLOCKΔ19 did not markedly alter the global autophagy as the ratio of LC3B-II/LC3B-I, a commonly used autophagy marker^38^, remained largely constant in transfected 293T cells (Supplementary Fig. 11).

Next, we measured BMAL1 half-life by CHX treatment with or without MG132 or 3-MA. In the absence of MG132 or 3-MA, ectopic Flag-CLOCK expression accelerated BMAL1 decay, whereas Flag-CLOCKΔ19 stabilized BMAL1 (Fig. 3b). Importantly, blocking either the proteasomal degradation (MG132) or autophagy (3-MA and CQ) markedly increased BMAL1 stability compared with mock and Flag-CLOCK transfection groups, suggesting functional involvement of both degradation pathways in BMAL1 turnover. Furthermore, in the presence of these inhibitors, BMAL1 displayed the highest levels with Flag-CLOCKΔ19 expression, in supportive of a stabilizing effect of CLOCKΔ19 on BMAL1. To further ascertain the regulatory roles of the two degradation pathways, we measured the steady-state levels of BMAL1 in the absence of CHX. Blocking either proteasomal degradation (MG132; Fig. 3c) or autophagy (3-MA or CQ; Fig. 3d,e) stabilized Flag-BMAL1, counteracting its degradation in the presence of Flag-CLOCK.

These observations collectively suggested that BMAL1 turnover is mediated via both proteasomal and autophagic pathways and that CLOCK and CLOCKΔ19 differentially modulate BMAL1 degradation.

### p62/SQSTM1 regulated CLOCK-induced BMAL1 degradation through autopahgy

**Figure 4:**
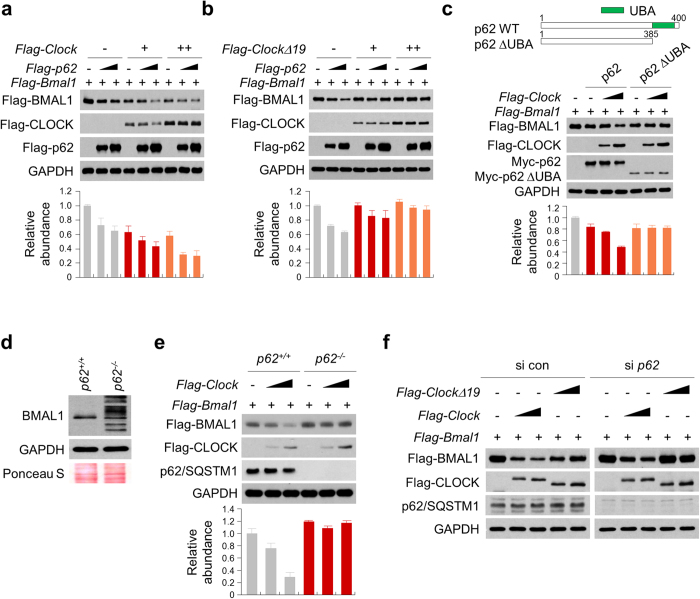
CLOCK and CLOCKΔ19 differentially regulated BMAL1 degradation in a p62-dependent manner. (**a**,**b**) 293T cells transfected with expression vectors for Flag-BMAL1, Flag-CLOCK, Flag-CLOCKΔ19, and Flag-p62 were lysed and subjected to immunoblotting analysis with antibodies against anti-Flag and GAPDH (loading control). (**c**) Schematic representation of p62 constructs employed were Myc-tagged WT p62; full-length p62 (amino acids 1 to 440), and p62 ΔUBA, a construct missing the UBA domain (amino acids 386 to 440) (upper panel). 293T cells transfected with expression vectors for Flag-BMAL1, Flag-CLOCK, Flag-CLOCKΔ19, and Myc-p62 (WT or ΔUBA) were lysed and subjected to immunoblotting analysis with antibodies against anti-Flag, anti-Myc and GAPDH (loading control). (**d**) Protein lysates were prepared from *p62*^+/+^ (WT) and *p62*^*−*/*−*^ MEF cells and subjected to immunoblotting analysis of BMAL1 and GAPDH. Ponseau S staining of the membrane following transfer was carried out as an additional loading control. (**e**) MEFs (*p62*^+/+^ or *p62*^*−*/*−*^) expressing Flag-BMAL1 and Flag-CLOCK were lysed and subjected to immunoblotting analysis with antibodies against anti-Flag, p62, and GAPDH (loading control). (**f**) 293T cells were transfected with indicated expression vectors in the presence of negative control or p62 siRNA for 48 h and then immunoblotted with the indicated antibodies. The relative levels of the targeted proteins were estimated by densitometry using the ImageJ program and the ratios were calculated relative to the GAPDH control. The data represent the mean ± SD of three independent experiments.

Autophagy is an important mechanism to prevent accumulation of polyubiquitinated protein aggregates unable to be degraded through the proteasomal pathway. In particular, the autophagy adaptor protein p62/SQSTM1 recognizes polyubiquitinated protein aggregates and incorporates them into autophagosomes via direct interaction with LC3-II on the autophagosomal membrane, thereby delivering the aggregates for degradation^39^. To directly investigate a role of p62 in the autophagic disposal of polyubiquitinated BMAL1, we co-transfected 293T cells with *Flag-Bmal1* and *Flag-p62* expression constructs in conjunction with either *Flag-Clock* (Fig. 4a) or *Flag-ClockΔ19* (Fig. 4b). Interestingly, ectopic expression of p62 enhanced BMAL1 degradation in a dose-dependent manner in the presence of CLOCK but not CLOCKΔ19, suggesting that the mutant form suppressed autophagic degradation of BMAL1.

p62/SQSTM1 binds to ubiquitin chains via the ubiquitin-associated (UBA) domain (Fig. 4c, top) respectively to deliver protein substrates to autophagosomes^39^. We next assessed the role of the p62 UBA domain in CLOCK-induced BMAL1 degradation using 293T cells expressing wild-type or UBA-deleted p62 (p62ΔUBA). Compared with the intact p62, p62ΔUBA failed to promote CLOCK-dependent BMAL1 degradation (Fig. 4c). The role of p62 was further investigated using autophagy defective, p62-deficient (*p62*^*−*/*−*^) MEF cells. In *p62*^*−*/*−*^ MEF cells, we observed elevated levels of endogenous BMAL1, including both higher molecular weight and truncated forms, compared with WT (Fig. 4d). Next to delineate the mechanism of CLOCK-dependent autophagic BMAL1 degradation, we transfected both wild-type (*p62*^+/+^) and *p62*^*−*/*−*^ MEF cells with *Flag-Bmal1* and *Flag-Clock*. As shown in Fig. 4e, the absence of p62 blocked Flag-BMLA1 degradation induced by CLOCK in *p62*^*−*/*−*^ cells. We next performed siRNA knockdown experiment to further ascertain a requisite role of p62 in CLOCK-induced BMAL1 degradation. In 293T cells treated with control siRNA, Flag-BMAL1 showed significant decreases in levels with Flag-CLOCK expression, and the decreases were much attenuated with Flag-CLOCKΔ19 (Fig. 4f, left). p62 knockdown showed robust protective effects on Flag-BMAL1, enhancing its levels significantly with greater degrees of enrichment observed with Flag-CLOCKΔ19 than Flag-CLOCK (Fig. 4f, right). Together, these results indicated an essential role of the cargo protein p62 in autophagy-dependent BMAL1 degradation.

### Insulin signaling was hyper-activated in HFD-fed Clk/+ mice

**Figure 5:**
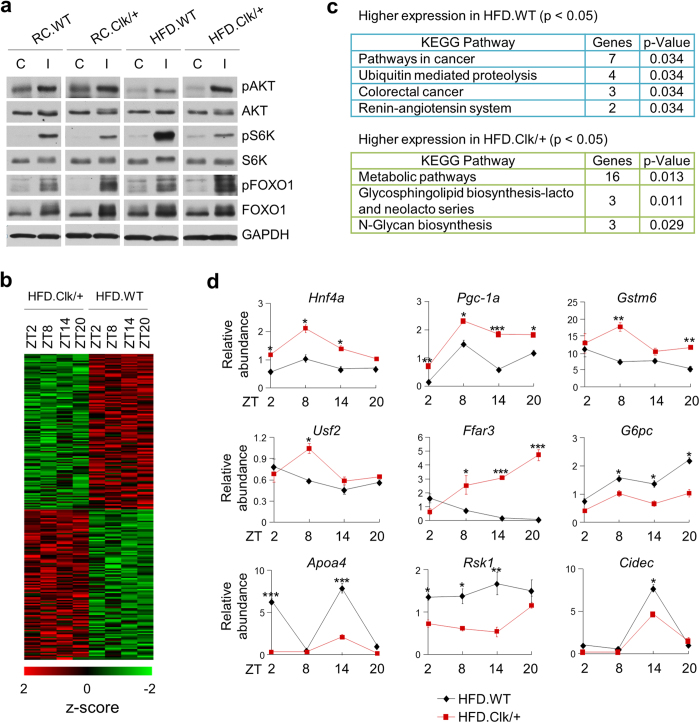
Clk/+ mice displayed reprogramming of metabolic signaling. (**a**) WT and Clk/+ mice were fed with RC or HFD for 12 weeks. Following overnight fasting, mice were subjected to insulin challenge and liver samples were collected. Immunoblotting of liver lysates was performed using the indicated antibodies against insulin signaling components. C and I denote vehicle control and insulin treatment respectively. (**b**) Microarray analysis was performed using total liver RNAs from HFD.WT and HFD.Clk/+ mice. Heat map for genes differentially expressed in HFD.WT and HFD.Clk/+ (p < 0.05) is shown. (**c**) KEGG pathway analysis of differentially expressed genes identified distinct pathways for HFD.WT and HFD.Clk/+ mice. (**d**) Real-time qPCR analysis of metabolic gene expression in HFD.WT and HFD.Clk/+ liver. Data was analyzed by two-way ANOVA, Bonferroni’s test. *p < 0.05; **p < 0.01; ***p < 0.001.

Growing evidence indicates an important role of the circadian clock, particularly BMAL1, in insulin signaling and energy homeostasis^20^,^22^. To understand the underlying mechanism of improved glucose homeostasis from HFD feed Clk/+ mice, we next conducted insulin challenge experiment using WT and Clk/+ mice under RC or HFD to further delineate insulin signaling response (Fig. 5a). As expected, acute insulin treatment of fasted mice led to elevated phosphorylated AKT (pAKT) levels, suggesting robust activation of insulin signaling. Importantly, greater levels of pAKT were found in HFD-fed Clk/+ mice relative to WT, whereas the total AKT protein levels remained largely unaltered. In contrast, pAKT induction was not altered between the genotypes under RC conditions. These results strongly indicated enhanced insulin signaling in Clk/+ mice with HFD feeding was responsible for the improved glucose homeostasis (Fig. 1a,c). In accordance with sensitized insulin signaling, we observed enrichment of phosphorylated FOXO1 (pFOXO1) and decreased levels of pS6K in Clk/+ mice relative to WT under both RC and HFD conditions. Importantly, however, the degrees of respective changes were markedly greater in HFD than in RC. FOXO1 is directly phosphorylated by AKT, and S6K is a direct target of mTOR that functions to negatively regulate insulin signaling^40^. These data together further indicated improved insulin signaling and glucose control in Clk/+ mice, particularly under HFD feeding conditions.

### Metabolic genes were differentially expressed in Clk/+ mice

Finally, to obtain a global view of the circadian transcriptome toward understanding the improved glucose homeostasis in Clk/+ mice compared with WT under HFD, we conducted circadian microarray transcriptome profiling (Affymetrix). Clock gene expression patterns (Supplementary Fig. 12) were largely concordant with the real-time qPCR results shown in Supplementary Fig. 6, serving as a quality control. Applying rigorous thresholds, we identified 114 and 110 transcripts (corresponding to 113 and 108 genes respectively) whose expression was up- or down-regulated by at least 1.2-fold in Clk/+ mice relative to WT under HFD (Fig. 5b; Supplementary Tables 1 and 2). Clock protein binding site analysis of gene bodies ±5 kb further revealed extensive clock protein binding to the promoters of differentially expressed genes (Supplementary Fig. 13). We next performed KEGG pathway analysis to gain functional insight on these genes. Interestingly, the top pathways associated with genes upregulated in Clk/+ were all metabolic in nature, including the top category with 16 genes (14%) specifically denoted as “Metabolic pathways” (Fig. 5c; Supplementary Table 2). Real-time qPCR analysis of a selected set of key metabolic genes further revealed significant expression changes in Clk/+ mice (Fig. 5d), including up-regulation of genes encoding liver transcription regulators HNF4α, PGC-1α and USF2 and conversely down-regulation of those encoding CIDEC, G6PC, and RSK1. Taken together, these data suggested beneficial metabolic gene reprogramming in the liver of Clk/+ mice fed with HFD.

## Discussion

In the current study, we observed an unexpected improvement in glycemic control in Clk/+ mice relative to WT under HFD feeding. Interestingly, molecular mechanistic studies revealed stabilization of BMAL1 by CLOCKΔ19 via a novel dual mechanism attenuating both proteasomal and autophagic degradation of BMAL1, providing the first evidence of autophagic regulation of a core clock component. Furthermore, we also found enhanced activation of insulin signaling components, as well as specific changes in metabolic gene expression, in HFD-fed ClK/+ mice relative to WT. Our findings reveal a novel mode of clock regulation by autophagy, and extend previous behavioral and cellular studies of Clk/+ circadian rhythms. Under normal RC conditions Clk/+ mice display persistent circadian wheel-running behavior characterized by both period lengthening and amplitude dampening^30^. Genetic suppressor studies revealed that melatonin signaling and the transcription factor USF1 can suppress period lengthening in Clk/+ mice^41^,^42^. Likewise, a group of clock-enhancing small molecules were able to restore the compromised oscillatory amplitude of Clk/+ reporter rhythms to WT levels^25^,^43^. Together with these previous genetic and pharmacological studies, our physiological and mechanistic studies illustrated remarkable plasticity of this partially impaired clock.

Our molecular analysis unveiled a novel mechanism for the enhanced accumulation of BMAL1 in Clk/+ mice involving bifurcated attenuation of both proteasomal and autophagic turnover. Consistent with previous findings^32^, we showed that MG132 block of proteasomal degradation led to elevated BMAL1 levels; furthermore, BMAL1 polyubiquitination was robust in the presence of CLOCK yet much diminished with CLOCKΔ19. We also provided multiple lines of evidence from pharmacological and genetic studies indicating a novel role of autophagy in BMAL1 turnover (see also below). Previous studies have shown circadian regulation of autophagy-related genes (Atgs)^36^,^37^; however, little was known how autophagy reciprocally regulates the clock. Our study thus provides the first molecular evidence for autophagic degradation of a core clock component in mammals.

In particular, our studies of BMAL1 K259R and the autophagy cargo protein p62 revealed molecular insight into the dual mechanism of proteasomal and autophagic degradation of poly-ubiquitinated BMAL1^34^. Interesting questions remain concerning the functional and mechanistic relationship between these BMAL1 disposal pathways. For example, it is not known whether under normal or pathological conditions they coordinate in any spatiotemporal manner to degrade distinct pools of BMAL1, such as cytoplasmic, nuclear or chromatin bound BMAL1. On the other hand, we demonstrated that BMAL1 ubiquitination and subsequent dual degradation were dependent on intact CLOCK proteins and conversely inhibited by CLOCKΔ19, providing a mechanistic explanation for the enhanced BMAL1 accumulation in the Clk/+ background. Our findings extend the previous observation of interdependent function and turnover of CLOCK and BMAL1^27^,^28^, and reveal exquisite clockwork mechanisms in both normal and partially compromised functional states. Of note, CRY1 levels were also enhanced in Clk/+ and Clk/Clk mice to varying degrees (Fig. 1g). CRYs have been shown to inhibit phosphorylation and subsequent transcriptional activation of CLOCK/BMAL1^44^,^45^. Given the coupling between CLOCK/BMAL turnover and transcriptional activity^27^,^32^,^46^,^47^, it will be interesting to determine whether CRY enrichment in the mutant mice also impinges on the dual attenuation mechanism to promote BMAL1 accumulation. Future studies will also delineate the detailed regulatory mechanism underlying the autophagic regulation of BMAL1 and the clock, and distinguish the functional roles of BMAL1 stabilization per se vs. the circadian clock in the observed metabolic improvement.

Clk/Clk mice have previously been shown to exhibit normal or enhanced insulin sensitivity at mid-age, under HFD or in the *ob*/*ob* genetic background^18^,^19^,^48^, yet pancreatic defects in insulin secretion elevated fasting glucose levels and compromised glucose tolerance^18^,^19^. In comparison, Clk/+ mice displayed improved glycemic control over WT with HFD feeding and also in the *db*/*db* mutant background, as evidenced by enhancement in both glucose tolerance and insulin tolerance under the conditions tested. It will be interesting to further determine these metabolic parameters as a function of circadian and fasting time. Given the important role of skeletal muscle and adipose tissues in systemic glucose metabolism^49^, future studies should also investigate extra-hepatic regulation of the improved glycemic control in Clk/+. The current work demonstrated that hepatic insulin signaling was preferentially activated in HFD.Clk/+ relative to HFD.WT, as phosphorylated AKT and its substrate FOXO1 were enriched whereas the negative factor phosphorylated S6K down-regulated. Moreover, we observed sustained circadian expression of BMAL1 in HFD.Clk/+ similar to that in RC conditions, consistent with previously reported functional connection between BMAL1 and insulin signaling. First, *Bmal1*^*−*/*−*^ mice were deficient in AKT activation in both liver and muscle and concomitantly suffered from impaired insulin sensitivity^22^. Furthermore, mTOR-S6K signaling was shown to be impaired as a result of BMAL1 deficiency *in vitro* and *in vivo*^50^. Finally, a recent study also illustrated a requisite role of *Bmal1* in lipogenesis regulation by the mTORC2-AKT signaling^51^.

We also observed that HFD feeding, while lengthening the free-running period in WT, did not show a similar effect in Clk/+ mice. This behavioral resistance to HFD-induced period lengthening in Clk/+ mice parallels the observed effects of HFD and CLOCKΔ19 on BMAL1 levels. Acute HFD intake has been reported to alter liver circadian clocks and temporal partitioning of food intake^52^, whereas chronic intake moderately lengthened the free-running period in WT mice^31^. In addition to its effect on BMAL1 abundance, HFD has also been reported to impact circadian transcription by regulating CLOCK/BMAL1 chromatin recruitment^53^. Of note, CLOCK/BMAL1 chromatin recruitment displayed circadian oscillation^54^, and transactivation of target genes coincided with depletion of these transcription factors^27^,^32^,^46^. Thus, the observed stabilization and enrichment of BMAL1 in HFD.Clk/+ may regulate circadian transcription at the steps of chromatin recruitment, promoter transactivation and subsequent CLOCK/BMAL1 degradation. For example, greater abundance of BMAL1 in Clk/+ may increase the likelihood of productive transcription complex formation with wild-type CLOCK proteins on target gene promoters, thus leading to elevated transcription. In that regard, it will be interesting to determine whether CLOCK and CLOCKΔ19 display differential BMAL1 binding affinity and kinetics to modulate BMAL1 level and transcriptional activity. Extending previous studies where HFD elicited a wide-spectrum *de novo* oscillation of circadian transcripts downstream from the core clock when compared with RC feeding^53^, our global transcriptome analysis revealed broad changes in levels of metabolic gene transcripts in HFD-fed Clk/+ compared with WT. These studies thus illustrate a dynamic plasticity in circadian and metabolic networks in response to the dietary challenge.

In conclusion, our study reveals unexpected behavioral and molecular remodeling in HFD-fed Clk/+ mutant mice as a model for a partially impaired clock. We provide evidence for a novel role of autophagy in CLOCK-dependent BMAL1 turnover, and illustrate activated insulin signaling concordant with improved glycemic control. Studies of partially impaired, yet still rescuable, clocks will yield rich insight into clock plasticity and interventional strategies.

## Materials and Methods

### Animals

Husbandry and experimental methods for all the animal experiments were in accordance with IACUC guidelines at the University of Texas Health Science Center at Houston (UTHSC-H), and the study was approved by UTHSC-H. Male wild-type (WT), *Clock*^*Δ19*/+^ (Clk/+), *Clock*^*Δ19*/*Δ19*^ (Clk/Clk), *db*/*db* (db:WT) and *db*/*db Clock*^*Δ19*/+^ (db:Clk/+) mice, all on the C57BL/6J genetic background, were obtained as littermates from heterozygous breeding using *Clock*^*Δ19*/+^^29^ and *db*/+ breeders (Jackson Laboratory) respectively. Mice were group-housed (2–4/cage) in a standard animal facility under a 12 h:12 h light:dark cycle. For circadian locomotor and metabolic chamber studies, mice were single-housed in an approved satellite facility.

### Reagents, plasmids and antibodies

Flag-Bmal1, HA-Bmal1, Flag-Clock and Flag-ClockΔ19 were subcloned into the pCMV10 3xFlag DNA vector (Sigma) for protein expression. Myc-p62 and Myc-p62ΔUBA mutant constructs were previously described^55^. Flag-p62 was subcloned into the pcDNA3.1 (Invitrogen). Site-directed mutagenesis of Bmal1 K259R construct clone was performed using the following primers.

Bmal1 K259R-F: TGCAACAGGCCTTCAGTACGGGTGGAAGATAAGGACTTC;

Bmal1 K259R-R: GAAGTCCTTATCTTCCACCCGTACTGAAGGCCTGTTGCA. All constructs were verified by sequencing. The primary antibodies used in the study are: Flag-tag (Sigma-Aldrich), Myc-tag, AKT, pAKT (Ser473), S6K, pS6K (Thr389) and FOXO1, pFOXO1 (Thr24) (Cell Signaling), BMAL1 (Cocalico, epitope: aa 381–579), CLOCK (Santa Cruz), LC3B (Novus), p62/SQSTM1 (Boster), REV-ERBα (Cell Signaling), PER2^56^, GADPH (Ambion), α-Tubulin (Santa Cruz) and Lamin B1 (Abcam). 3-methyladenine, MG132, chloroquine, and cycloheximide were purchased from Sigma-Aldrich.

### Mouse diets and body weight measurements

WT and Clk/+ mice at 6 weeks of age were fed with regular chow diet (Purina 5001) or HFD (D12492, Research Diets) until the end of the experimental protocol. Weekly body weight was monitored in WT and Clk/+ mice for 12–14 weeks. For db:WT and db:Clk/+ mice, mice at 6–8 weeks of age were group-housed (2–3/cage) and maintained on RC.

### Energy expenditure and food intake measurements

Energy expenditure was examined by measuring oxygen consumption with indirect calorimetry as described^57^. After 10 weeks of treatments described above, mice from each group were placed at room temperature (22–24 °C) in the chambers of a Comprehensive Lab Animal Monitoring System (CLAMS, Columbus Instruments). After mice adapted to the metabolic chamber, volume of O_2_ consumption and CO_2_ production was continuously recorded over a 24 h period. Average O_2_ consumption was calculated and compared between different treatments. Food and water were provided *ad libitum*. To measure food intake, food pellets were weighted every three hours over a 24 h period in WT and Clk/+ mice fed with RC or HFD. The daily food intake was calculated from averaged food intake of three independent experiments.

### Serum triglyceride, cholesterol, free fatty acid and insulin assays

Serum samples were obtained at ZT2 from treated WT and Clk/+ mice as mentioned above. Serum triglyceride, cholesterol and free fatty acid levels were assessed by Serum Triglyceride Determination Kit (Sigma), Cholesterol Assay Kit (Cayman), and Free Fatty Acid Quantitation kit (BioVision) respectively. Serum insulin levels were detected with the Rat/Mouse Insulin ELISA kit (Millipore) according to the manufacturer’s instructions. The assay plates were read by a TECAN M200 instrument (Tecan).

### Glucose tolerance test, insulin tolerance test and insulin challenge assays

Glucose tolerance test (GTT) and insulin tolerance test (ITT) in all groups were performed largely as described^58^. Briefly, after overnight and 5-h fasting, GTT and ITT were conducted at ZT2 and ZT 8 respectively. Glucose levels were measured from tail blood before and 15, 30, 60, or 120 min after i.p. injection of either 1 g/kg glucose or 0.75 U/kg insulin (Eli Lilly) by using the ONETOUCH glucometer (LifeScan).

For insulin challenge, mice were given vehicle (PBS) or insulin (5 U/kg) via i.p following overnight fasting (15–18 h). Five minutes after injection, mice were deeply anesthetized by isoflurane inhalation. Isolated liver samples were immediately frozen in liquid nitrogen and stored in −80 °C freezer until later use.

### Circadian locomotor activity

After 12 weeks feeding of RC or HFD, WT and Clk/+ mice were used for circadian locomoter activity experiments. Briefly, mice were first maintained for 2 weeks in a 12 h:12 h light:dark (LD) cycle, then released into the constant darkness, free-running condition with *ad libitum* feeding for another 2 weeks. Wheel-running data was downloaded as VitalView data files and analyzed with the ActiView program^59^.

### Quantitative RT-PCR (qRT-PCR)

Total RNA was extracted from cells using the *PureXtract* RNAsol reagent (GenDEPOT) following the manufacturer’s protocol. cDNA was synthesized from 2 μg of total RNA using *amfiRivert* Platinum cDNA synthesis kit (GenDEPOT). Specific qRT-PCR primers are listed in sTable 3. qRT-PCR was performed using a MaxPro3000 Thermocycler (Agilent) with *amfiSure* qGreen qPCR Master Mix (GenDEPOT). Expression of the target gene was analyzed by a relative amount quantification method (ddCt) and normalized using *Gapdh* levels.

### Western blotting analysis

The Western blotting, or immunoblotting, procedures have been previously described^3^. Briefly, the cells were washed twice with cold PBS on ice and harvested by scraping with a rubber policeman. The cells were pelleted by centrifugation at 4 °C and resuspended directly into a lysis buffer plus protease inhibitor cocktail (GenDEPOT). Immunoblotting using mouse liver tissues was performed as previously described^3^. Densitometric analysis was performed using the ImageJ software to quantify the entire band regions with proper background subtraction. Protein half-life was calculated by using GraphPad Prism via nonlinear, one-phase exponential decay analysis.

### Cell culture and transfection

Adult mouse ear fibroblast and mouse embryonic fibroblast (MEF) cells were previously described^25^. Fibroblast cells and 293T cells were cultured in Dulbecco’s modified Eagle’s medium (DMEM, GenDEPOT) supplemented with 10% fetal bovine serum (FBS). For plasmids transfection, the cells were transfected with indicated plasmids using either iMFectin Poly DNA transfection reagent (GenDEPOT) or Lipofectamine 2000 (Invitrogen) for 24 h. For siRNA treatment, cultured 293T cells were transfected with p62/SQSTM1 siRNA (Santa Cruz) or a RNA interference negative control (Santa Cruz). Each well was incubated for 48 h with 10 nmol using Lipofectamine 2000. For inhibitor treatment, 293T cells transfected with expression vectors for indicated constructs were incubated in the absence or presence of MG132 (10 μM), 3-methyladenine (3-MA, 5 mM for MEF cells and 1 mM for 293T) or chloroquine (CQ, 25 μM), respectively. For palmitate treatment, cells were split into 60 mm dishes at an initial density of 3 × 10^5^ cells and incubated for 2–4 days (100 nM Dex) followed by 50 μM sodium palmitate treatment for 24–36 h before synchronization.

### Cytoplasm/nuclear fractionation

Nuclear and cytoplasmic fractionation was performed as previously described^3^. Briefly, 293T cells were washed with PBS and resuspended in 5 volumes of cell pellets with Buffer A and incubated on ice for 10 min. 10% Triton X-100 was added to the final concentration of 0.1% and vortexes for 15 sec. Cells were centrifuged at 4,000 rpm for 5 min to obtain the supernatants (cytoplasm), and the pellets (nucleus) were washed once with Buffer A. The pellets were subsequently resuspended in cell lysis buffer and centrifuged at 13,000 rpm for 30 min at 4 °C.

### Ubiquitination assay

293T cells were transiently transfected with indicated plasmids and treated with 10 μM MG132 for 1 h before harvesting. Cells were lysed with lysis buffer containing protease and deubiquitinase inhibitors and immunoprecipitated with anti-Flag antibody. The samples were subjected to SDS-PAGE followed by Western blotting. Ubiquitinated BMAL1 was detected by BMAL1 antibody.

### Microarray analysis

Total RNAs prepared from liver tissues from HFD-fed WT and Clk/+ mice was reverse transcribed into cDNAs, which were then biotin-UTP labeled and hybridized to the Illumina mouse WG-8v2.0 Expression BeadChip. Background was subtracted and arrays were normalized using quantile. To determine differentially expressed genes, genes with lowest detection p > 0.05 were filtered out. The mean expression level across the time course was compared between WT and Clk/+, and genes with statistically significant (t-test, p < 0.05) and at least 1.2 fold change were determined. Heat maps were generated by MultiExperiment Viewer. The WebGestalt program (http://www.webgestalt.org) was used for KEGG pathway analyses. ChIP-seq data^8^,^60^ was used to determine the number of the clock proteins binding sites within the gene body ±5 kb.

### Statistical analysis

Unless otherwise stated, results are presented as mean ± SEM. Data were analyzed using Student’s t-test, one-way ANOVA followed by post-hoc analysis using Dunnett’s multiple comparison tests or two-way ANOVA followed by post hoc analysis using Bonferroni test as appropriate. A value of p < 0.05 was considered statistically significant.

## Additional Information

**How to cite this article**: Jeong, K. *et al.* Dual attenuation of proteasomal and autophagic BMAL1 degradation in *Clock*^∆^^1^^9/+^ mice contributes to improved glucose homeostasis. *Sci. Rep.* **5**, 12801; doi: 10.1038/srep12801 (2015).

## Supplementary Material

Supplementary Information
