# Supplementary material for: Dual attenuation of proteasomal and autophagic BMAL1 degradation in ClockΔ19/+ mice contributes to improved glucose homeostasis
Source: Sci Rep. 2015 Jul 31;5:12801. doi: 10.1038/srep12801 (PMC4521189; doi:10.1038/srep12801)
Supplement: Supplementary Information [file srep12801-s1.pdf]

Supplementary Information

Dual attenuation of proteasomal and autophagic BMAL1 degradation in *Clock*$^{\Delta 19/+}$ mice contributes to improved glucose homeostasis

Kwon Jeong[1*], Baokun He[1*], Kazunari Nohara[1], Noheon Park[2], Youngmin Shin[1], Seonghwa Kim[1], Kazuhiro Shimomura[3], Nobuya Koike[4], Seung-Hee Yoo[1], Zheng Chen[1]

1. Department of Biochemistry and Molecular Biology, The University of Texas Health Science Center at Houston, 6431 Fannin St., Houston, TX 77030
2. Department of Neuroscience, The University of Texas Southwestern Medical Center, 5323 Harry Hines Blvd., Dallas, TX 75390
3. Department of Neurobiology and Physiology, Center for Sleep and Circadian Biology, Northwestern University, 2205 Tech Drive, Evanston, IL 60201
4. Department of Physiology and Systems Bioscience, Kyoto Prefectural University of Medicine, Kyoto, 602-8566, Japan

*: These authors contributed equally.

Correspondence should be addressed to Zheng Chen, Department of Biochemistry and Molecular Biology, The University of Texas Health Science Center at Houston, 6431 Fannin Street, MSB 6.200, Houston, TX 77030, USA. Tel.: 713-500-6284; Fax: 713-500-0652; E-mail: Zheng.chen.1@uth.tmc.edu .

This file contains 13 Supplementary Figures and 3 Supplementary Tables.

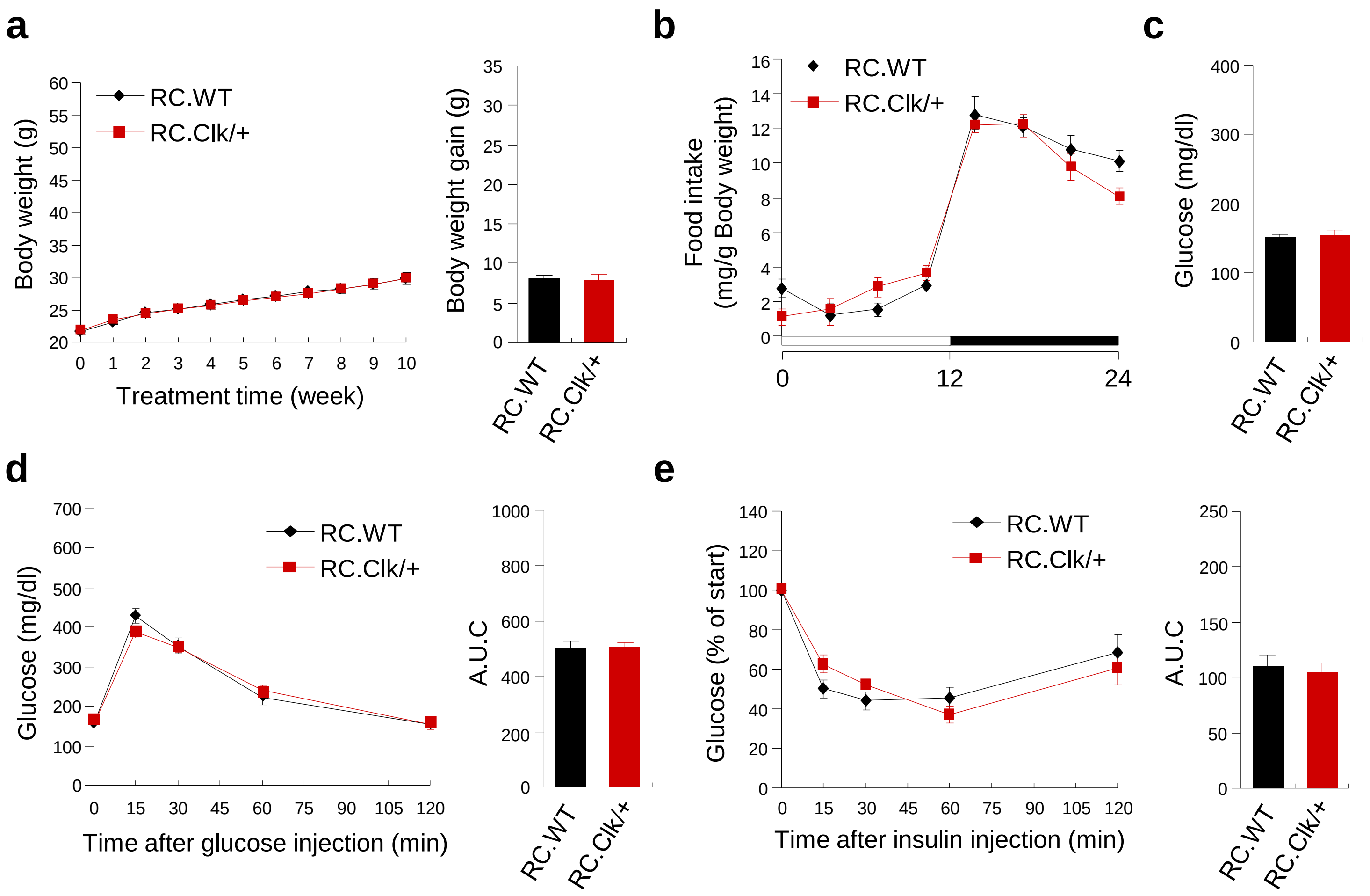

Supplementary Fig. 1. Under regular chow (RC) feeding Clock$^{\Delta 19/+}$ (Clk/+) mutant mice showed similar energy homeostasis with WT mice. (**a**) Average body weight of WT and Clk/+ mice fed with RC for 10 weeks (n=6-8). (**b**) Diurnal rhythm of food intake in RC-fed WT and Clk/+ mice (n=3). (**c-e**) Fasting glucose levels (**c**), glucose tolerance test (GTT) (**d**) and insulin tolerance test (ITT) (**e**) in WT and Clk/+ mice fed with RC for 10 weeks (n=6-8). Area under curve (A.U.C) data are shown as right panels in (**d**) and (**e**). Data are presented as mean ± SEM. One-way ANOVA was performed with SigmaStat.

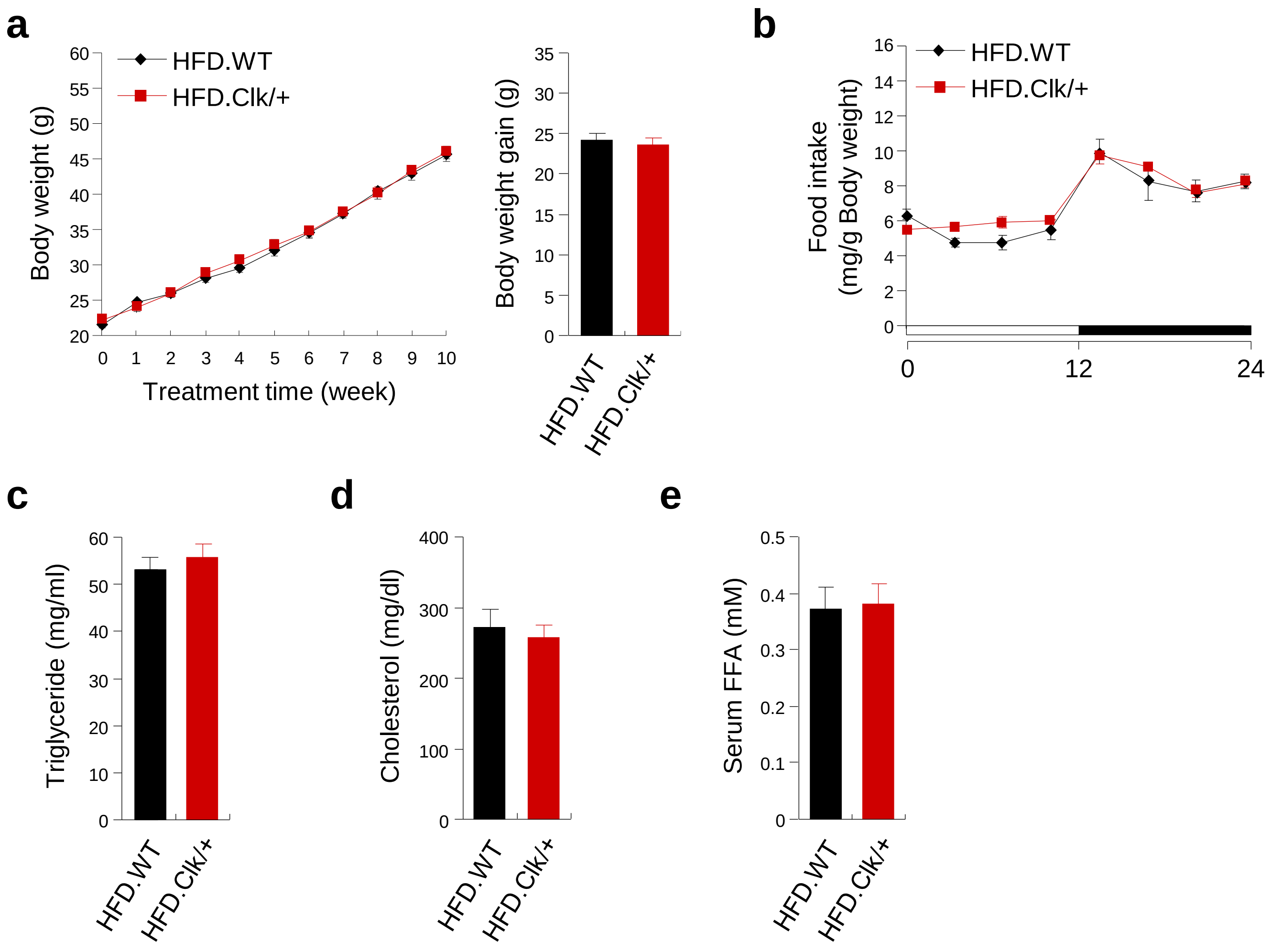

Supplementary Fig. 2. Clk/+ and WT mice showed similar body weight gain, food intake and serum lipids with high-fat diet (HFD) feeding. (**a**) Average body weight (left) and body weight gain (right) of WT and Clk/+ mice fed with HFD for 10 weeks (n=8). (**b**) Diurnal rhythm of food intake in HFD-fed WT and Clk/+ mice (n=3). (**c,d,e**) Serum triglyceride (**c**), cholesterol (**d**), and free fatty acid (**e**) levels in WT and Clk/+ fed with HFD mice for 10 weeks (n=6-15). Data are presented as mean ± SEM. One-way ANOVA was performed with SigmaStat.

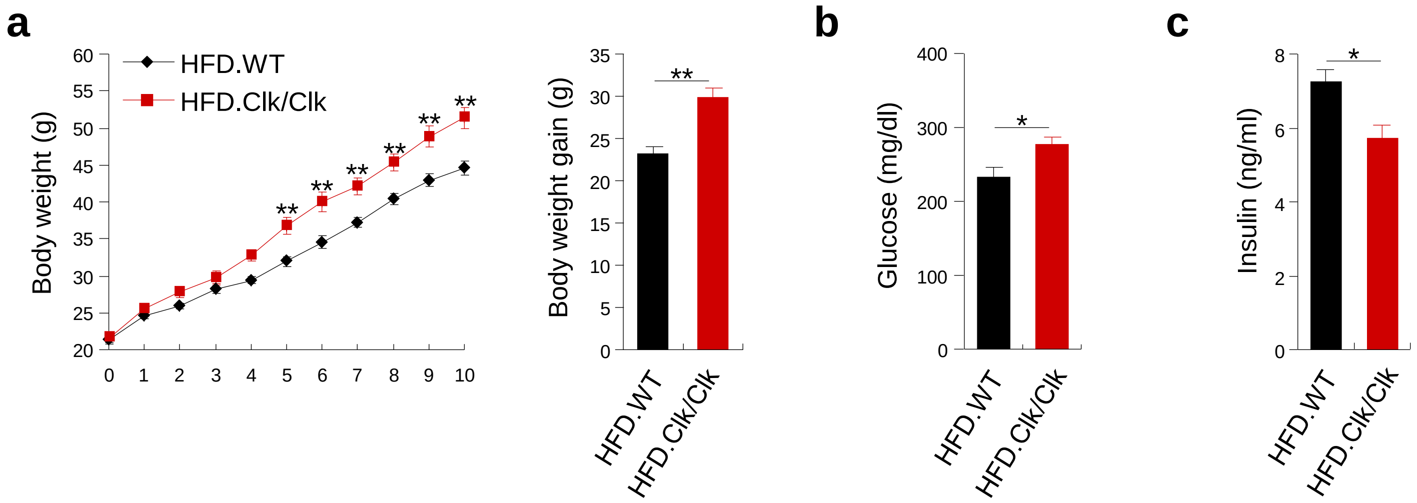

Supplementary Fig. 3. Clock$^{\Delta19/\Delta19}$ (Clk/Clk) mutant mice showed disrupted energy homeostasis with HFD feeding. Average body weight (**a**), fasting glucose (**b**) and insulin level (**c**) in WT and Clk/Clk mice fed with HFD for 10 weeks (n=6-11). Data are presented as means ± SEM. *p<0.05, **p<0.01. One-way ANOVA was performed with SigmaStat.

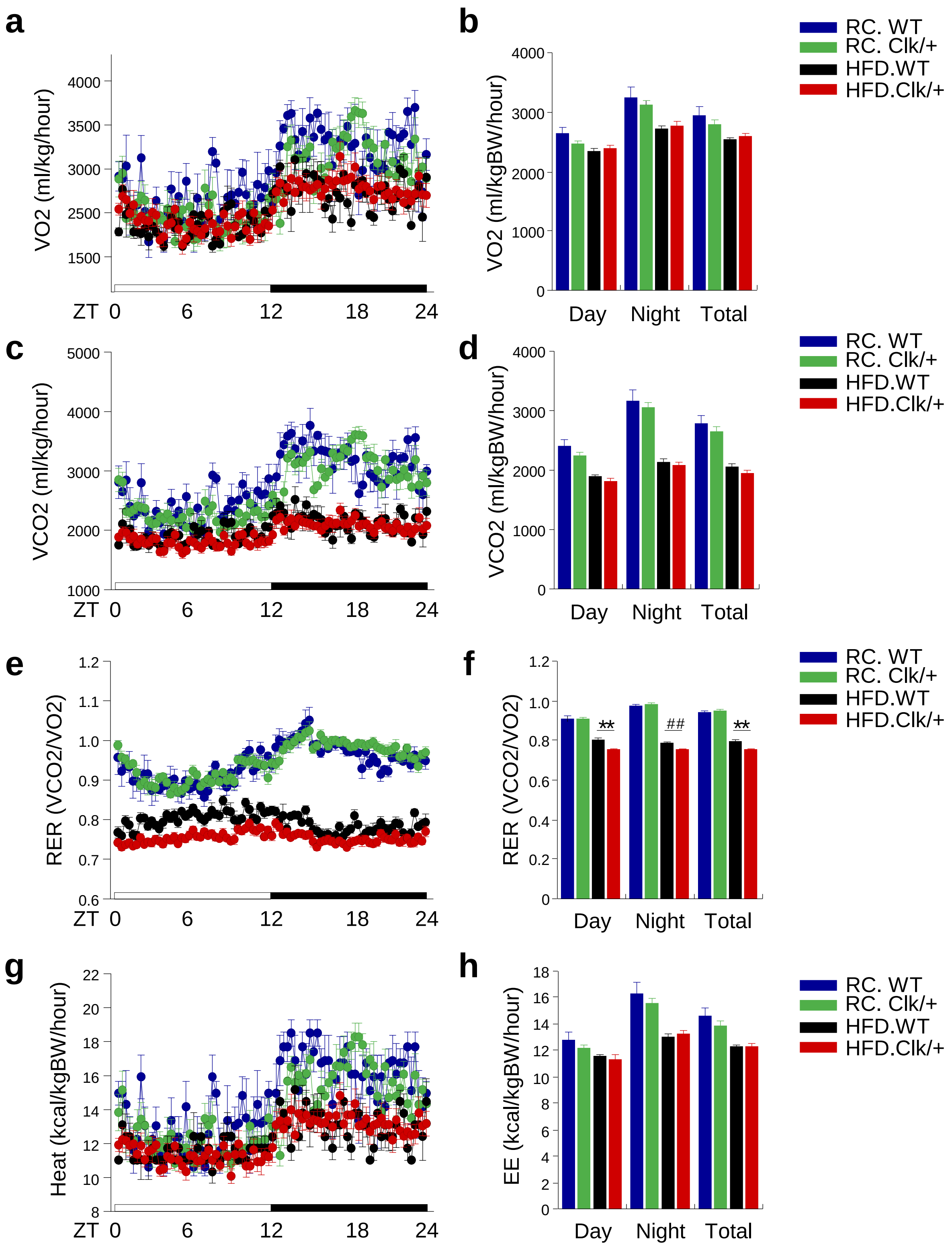

Supplementary Fig. 4. Daily metabolic rhythm measured by metabolic chambers. Daily rhythms of VO2 (**a**), VCO2 (**c**), RER (**e**) and Heat production (**g**). (**b,d,f,h**) Quantification of daytime (ZT0-ZT12), nighttime (ZT12-ZT24) and total (ZT0-ZT24) data from (**a**), (**c**), (**e**) and (**g**) are shown. **p<0.01, one-way ANOVA; ##p<0.01, student's t-test. Note the significant dampening effects of HFD on metabolic rhythmicity.

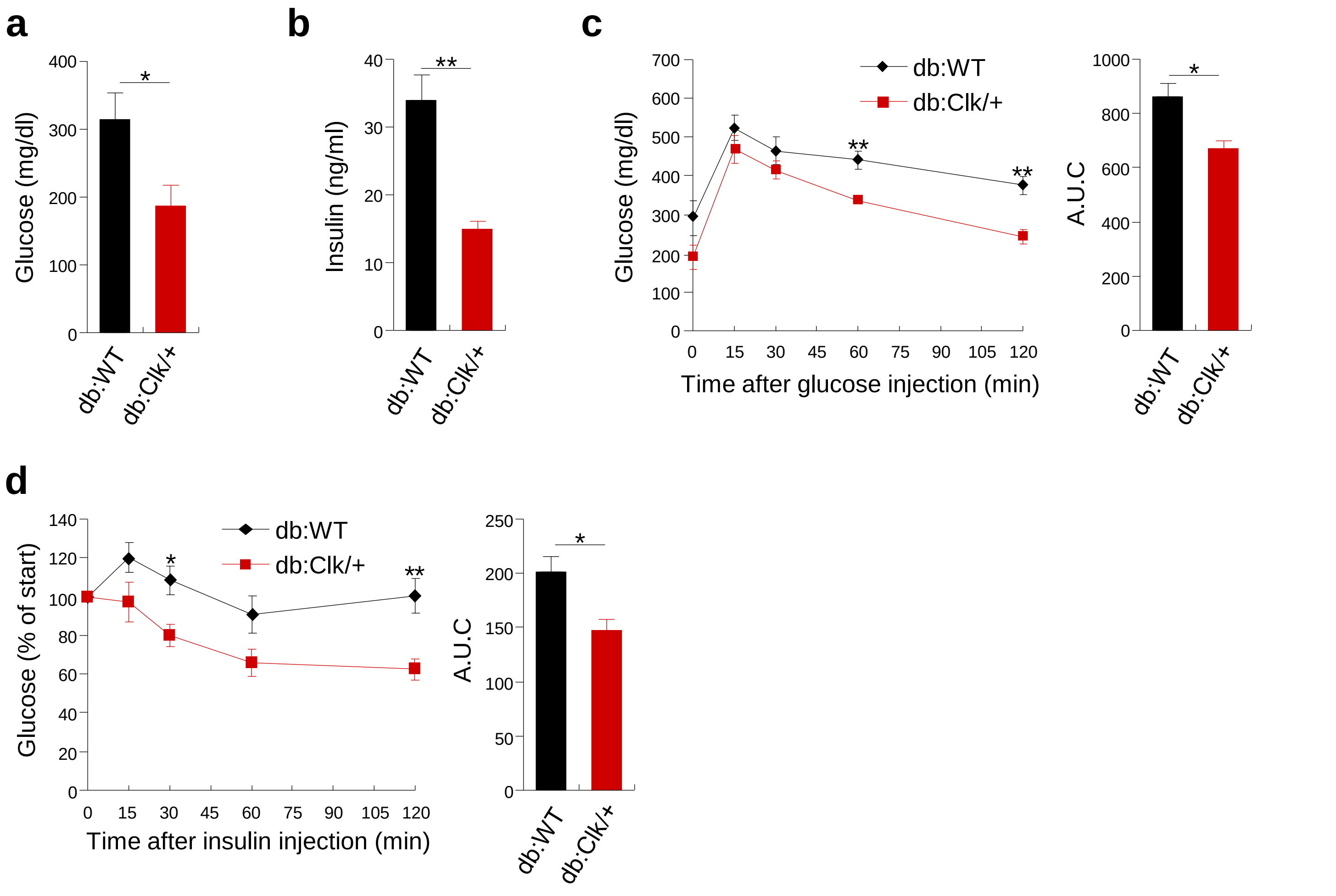

Supplementary Fig. 5. Clk/+ mutant in the *db/db* genetic background (denoted as db) showed improved glucose homeostasis. Fasting glucose levels (**a**), blood insulin levels (**b**), glucose tolerance test (GTT) (**c**) and insulin tolerance test (ITT) (**d**) in db:WT (denoting *db/db* WT) and db:Clk/+ (denoting *db/db Clk/+*) mutant mice (n=5-6). Area under curve (A.U.C) quantitation is shown for (**c**) and (**d**). Values are presented as means ± SEM. *p<0.05, **p<0.01. One-way ANOVA was performed with SigmaStat.

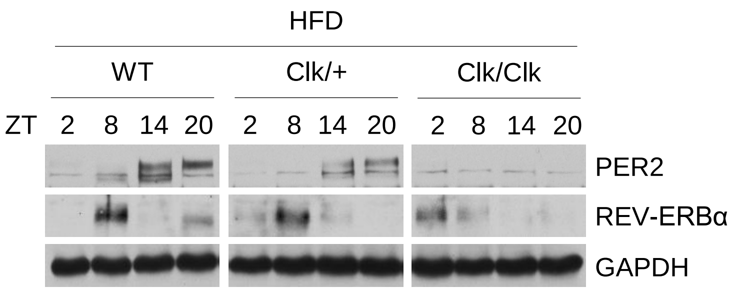

Supplementary Fig. 6. Immunoblotting analysis of PER2 and REV-ERBα proteins. Liver tissues from HFD fed WT, Clk/+ and Clk/Clk as described in Fig. 1g were used. GAPDH serves as the loading control.

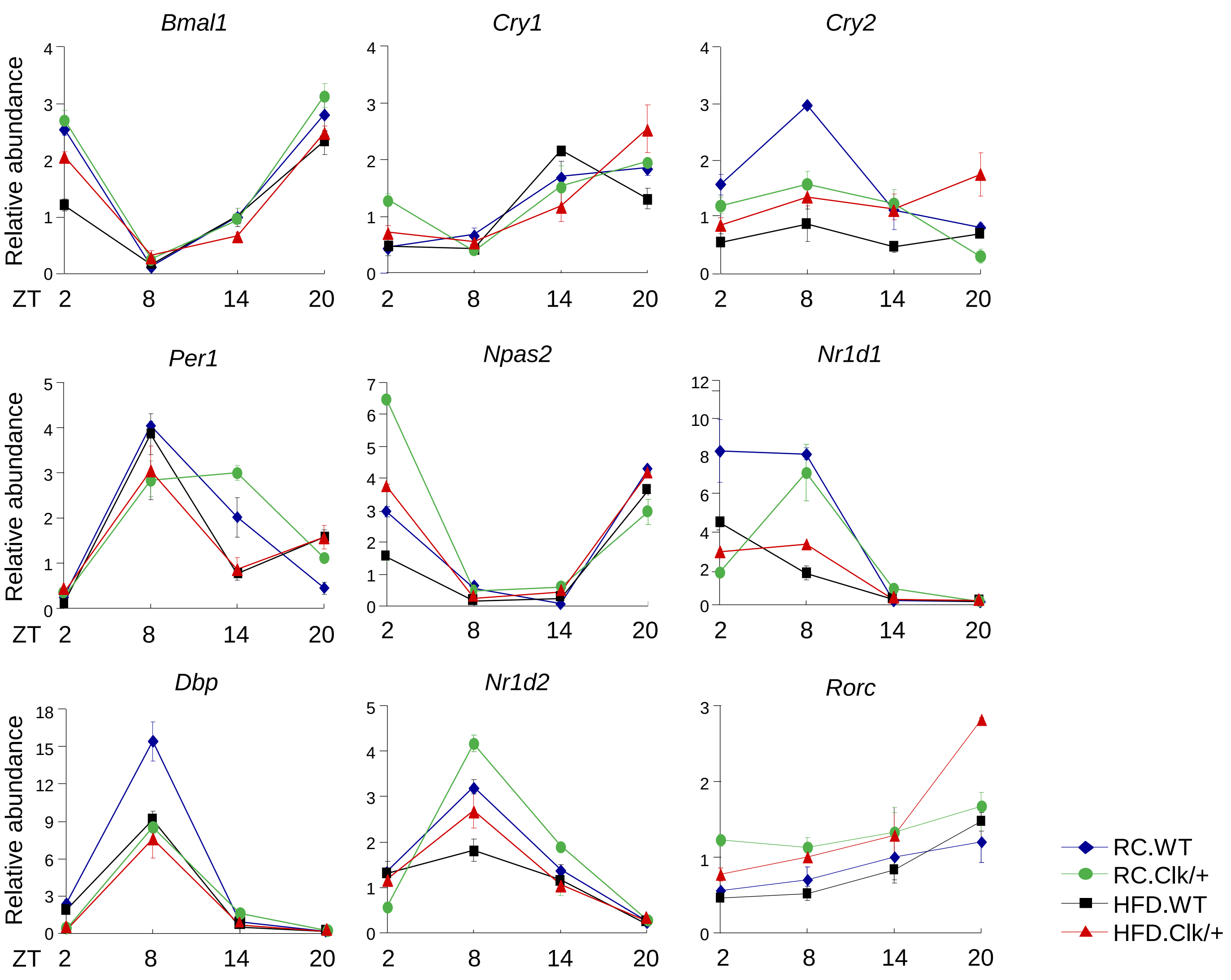

Supplementary Fig. 7. Real-time qPCR analysis of core clock genes in livers from WT and Clk/+ mice fed with RC and HFD. Data are presented as mean ± SD. Two-way ANOVA with Bonferroni *post-hoc* tests shows no significant statistical differences between RC.WT and RC.Clk/+, whereas significant statistical difference was detected between HFD.WT and HFD.Clk/+ (p<0.001) in *Bmal1* and *Cry1* expression. Expression of *Npas2* and *Nr1d2* in both RC.Clk/+ and HFD.Clk/+ mice was significantly increased compared with RC.WT and HFD.WT respectively (p<0.0001). p<0.0001, *Cry2* and *Dbp,* RC.WT vs RC.Clk/+.

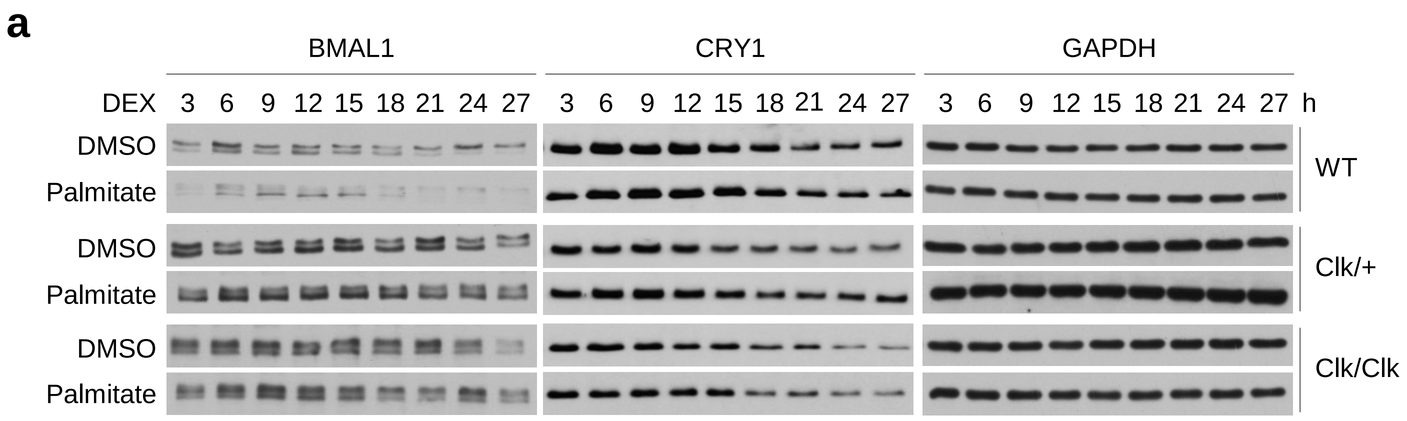

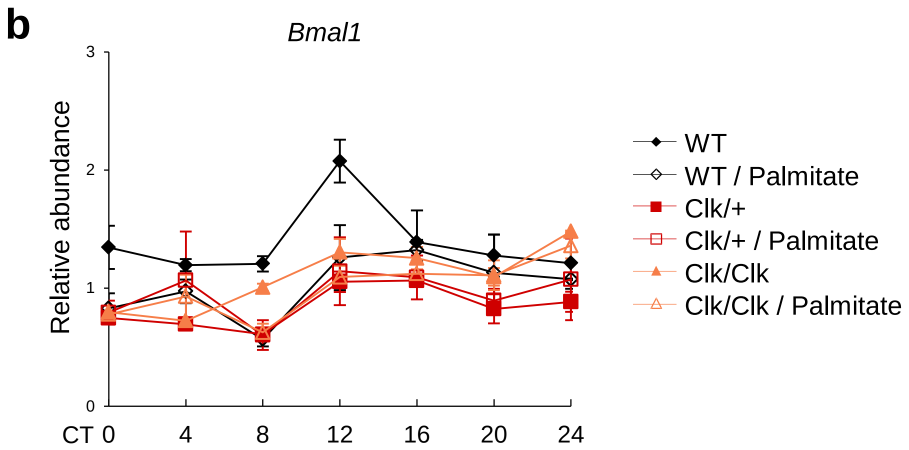

Supplementary Fig. 8. Effect of palmitate treatment on Bmal1 protein and transcript expression in WT, Clk/+ and Clk/Clk MEFs cells. (**a**) Immunoblotting analysis of BMAL1 and CRY1 proteins, and (**b**) Real-time qPCR analysis of *Bmal1* transcript levels in WT, Clk/+ and Clk/Clk MEFs cells. Two-way ANOVA shows significant differences between WT and WT/Palmitate group ($p<0.0001$).

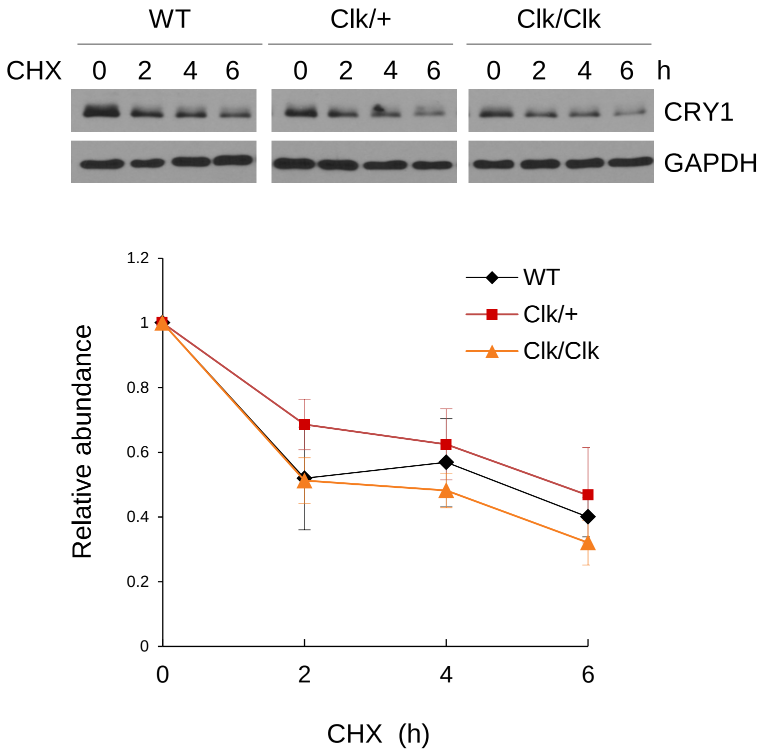

Supplementary Fig. 9. Effect of CLOCKΔ19 on CRY1 protein stability. WT, Clk/+ and Clk/Clk MEF cells were exposed to cycloheximide (CHX; 25 μg/ml) for the indicated times, and BMAL1 levels were assessed by immunoblotting analysis. Quantification is presented in the lower panel. Error bars represent mean ± SD (n=3). Half-life was determined by using nonlinear, one-phase exponential decay analysis, WT: 4.4 h, Clk/+: 5.5 h, Clk/Clk: 3.5 h.

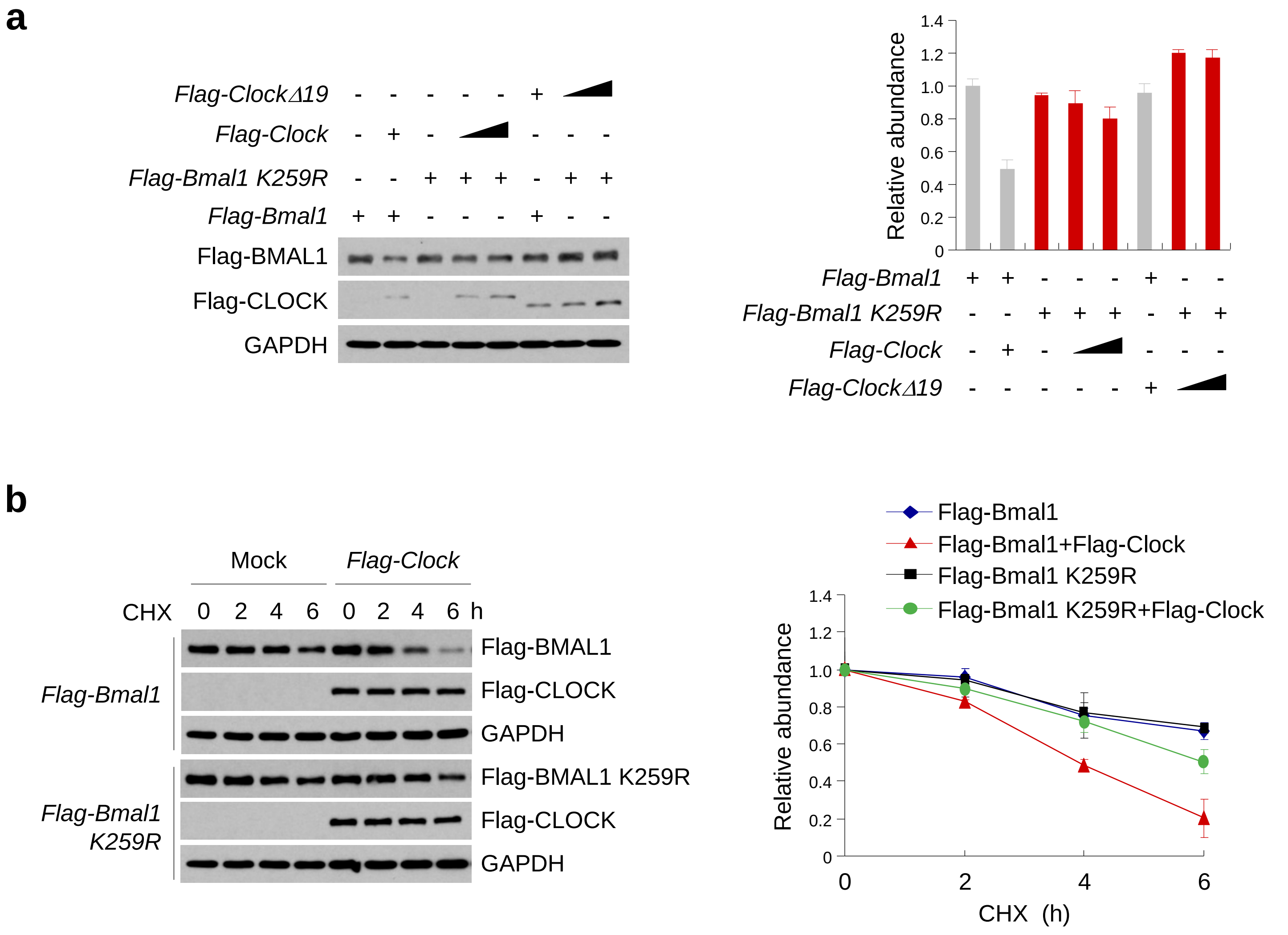

Supplementary Fig. 10. The K259R mutation in BMAL1 abolished effects of CLOCK on BMAL1 stability. (**a**) 293T cells transfected with expression vectors for Flag-BMAL1, Flag-BMAL1 K259R, Flag-CLOCK, and Flag-CLOCKΔ19 were lysed and subjected to immunoblotting analysis with antibodies against Flag and GAPDH (loading control). (**b**) 293T cells transfected with expression vectors for Flag-BMAL1, Flag-BMAL1 K259R, and Flag-CLOCK. These cells were exposed to CHX (25 µg/ml) for the indicated times. BMAL1 levels were assessed by immunoblotting at the indicated times after CHX treatment. Right panel: Quantification of the effects of CLOCK on BMAL1 and BMAL1 K259R stability. Error bars represent mean ± SD (n=3). Half-life was determined by using nonlinear, one-phase exponential decay analysis.

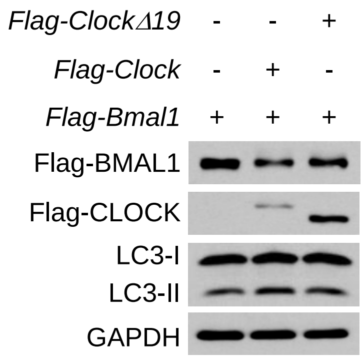

Supplementary Fig. 11. Ectopic expression of BMAL1, CLOCK and CLOCKΔ19 showed no significant effects on LC3-II level. 293T cells transfected with expression vectors for Flag-BMAL1, Flag-CLOCK, and Flag-CLOCKΔ19 were lysed and subjected to immunoblotting analysis with antibodies against Flag, LC3B, and GAPDH (loading control).

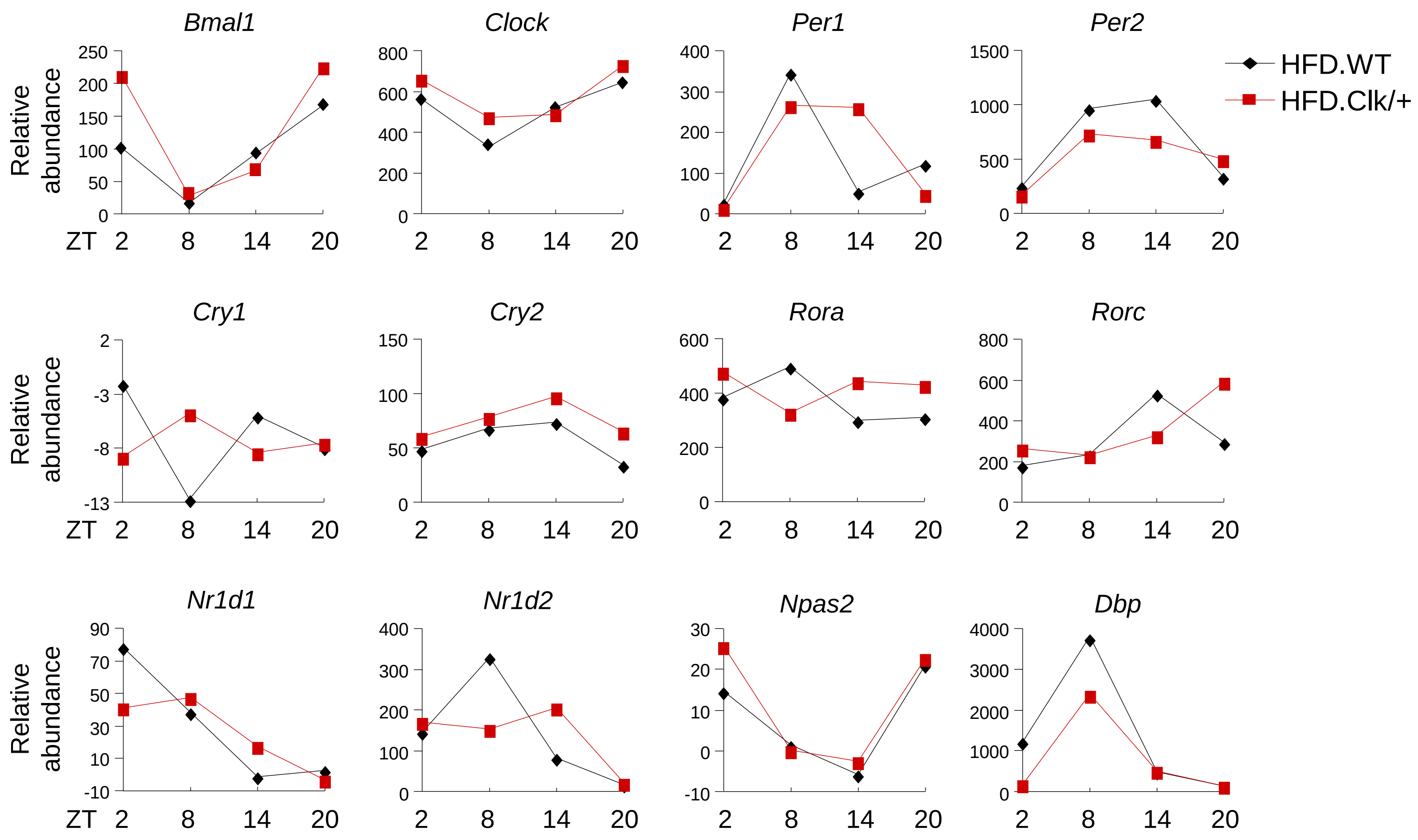

Supplementary Fig. 12. Expression of core clock genes in the liver of HFD.WT and HFD.Clk/+ mice by microarray analysis. The mRNAs from liver samples in HFD.Clk/+ and HFD.WTmice collected over the circadian cycle were subjected to microarray analysis. Relative abundance for each transcripts is shown.

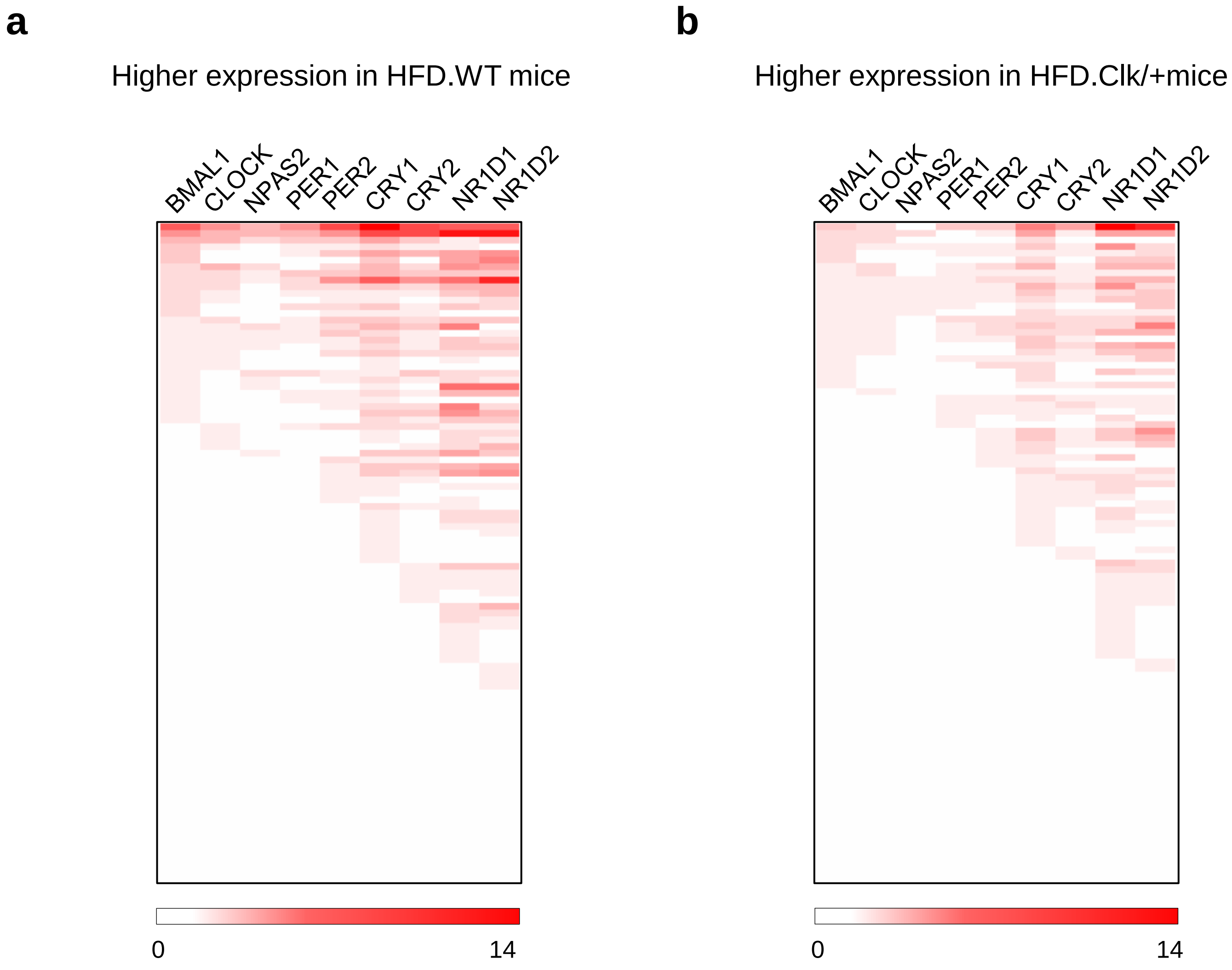

Supplementary Fig. 13. Heat maps of clock protein binding on the promoters of genes with higher expression levels in HFD.WT (a) or HFD.Clk/+ (b). Analysis was based on published ChIP-Seq results (Koike et al., 2012; Cho et al., 2012). The red scale shows the extent of binding as quantified by enrichment of immunoprecipitated DNA fragments.

Supplementary Table 1. List of liver genes down-regulated in HFD.Clk/+ relative to HFD.WT mice (fold change ⩾1.20) as identified by microarray analysis.

Gene	Fold Change	p-Value
TRIM52	86.50	0.049
1110054O05RIK	48.00	0.023
ASB7	42.29	0.039
TGFBR2	26.11	0.043
D3BWG0562E	19.00	0.043
1700109H08RIK	13.90	0.026
GPS1	7.00	0.041
GLOD4	6.58	0.027
ARID4A	6.48	0.029
ST6GALNAC4	6.06	0.006
CDC6	4.31	0.048
RNF208	3.95	0.008
BC028528	3.86	0.021
DUSP6	3.13	0.004
TM4SF4	3.10	0.040
DFNA5H	2.84	0.038
PHF12	2.81	0.040
COL27A1	2.71	0.018
RNF26	2.70	0.007
EPHA7	2.65	0.024
SMPD3	2.62	0.034
MICAL2	2.59	0.006
SAMD4	2.56	0.038
ERDR1	2.51	0.009
CML4	2.39	0.034
RIPK3	2.31	0.011
GM1568	2.30	0.046
LOC100045188	2.29	0.006
BIRC5	2.24	0.041
BC016423	2.20	0.005
KLC2	2.06	0.013
CSF1	2.03	0.042
OLFR497	2.02	0.026
GIT1	2.01	0.033
SMURF1	1.96	0.039
RFTN2	1.93	0.046
1110004E09RIK	1.92	0.025
SYNM	1.88	0.025
MME	1.88	0.037
DVL1	1.88	0.025
BRE	1.85	0.023
AIFM2	1.82	0.009

SH3D19	1.79	0.035
VGLL2	1.79	0.023
1200011M11RIK	1.78	0.044
GRIN2C	1.78	0.043
SERPINA3H	1.74	0.024
GPC1	1.72	0.015
1110048D14RIK	1.72	0.013
C1QTNF3	1.69	0.010
LIG4	1.69	0.039
HDAC6	1.68	0.041
PDZD8	1.68	0.019
NLN	1.68	0.036
CLEC1A	1.67	0.042
GTPBP5	1.65	0.040
PAPLN	1.64	0.014
CCDC49	1.64	0.015
PAM	1.63	0.050
SLC25A25	1.62	0.030
HPCAL1	1.61	0.048
TCFCP2	1.58	0.028
CCND1	1.56	0.028
SMPD4	1.55	0.026
HABP4	1.53	0.021
ZGPAT	1.51	0.028
CRIM2	1.50	0.030
BC057893	1.50	0.017
ENO3	1.49	0.011
DST	1.49	0.012
ARHGAP25	1.49	0.031
PAQR7	1.48	0.015
GTPBP6	1.47	0.008
TRIP6	1.46	0.023
ST3GAL3	1.44	0.048
RND3	1.43	0.043
EMILIN1	1.43	0.029
ARL2BP	1.41	0.043
POM121	1.41	0.017
MLKL	1.41	0.007
SMU1	1.41	0.029
MTMR14	1.40	0.048
TRIB2	1.40	0.027
NCDN	1.39	0.019
NCKIPSD	1.38	0.034
L3MBTL3	1.38	0.038
OASL1	1.38	0.020
4933407P14RIK	1.37	0.039
CCND1	1.37	0.027

CUL2	1.36	0.025
3110082I17RIK	1.36	0.044
SWAP70	1.36	0.019
IFIT3	1.35	0.049
HSD17B11	1.35	0.021
DUSP6	1.34	0.033
PTPLB	1.33	0.031
RAB3A	1.32	0.024
TSEN2	1.32	0.020
SKP2	1.30	0.039
RIOK1	1.29	0.019
SRF	1.28	0.018
1190005I06RIK	1.27	0.029
ENTPD5	1.26	0.029
D6WSU176E	1.25	0.018
THOC5	1.24	0.002
PIAS2	1.24	0.012
BLVRB	1.23	0.020
TPI1	1.22	0.044
SNAPC3	1.22	0.034
IPO5	1.22	0.048

Supplementary Table 2. List of liver genes up-regulated in HFD.Clk/+ over HFD.WT mice (fold change ⩾1.20) as identified by microarray analysis. The genes marked in red are denoted by KEGG in the "Metabolic pathways".

Gene	Fold Change	p-Value
0610012A05RIK	78.75	0.045
FFAR3	51.56	0.004
NR5A1	36.11	0.009
FXYD2	24.11	0.031
LOC225594	10.67	0.016
PIK3R6	10.58	0.037
TCFAP2A	10.39	0.021
FUT7	10.35	0.030
4933429E10RIK	10.25	0.028
ITGB6	6.81	0.026
9130401M01RIK	4.71	0.014
ADAT1	3.79	0.027
HIST1H2AA	3.67	0.044
B3GALT5	3.65	0.047
COL11A2	3.22	0.009
KRT78	3.03	0.045
SHC1	2.76	0.030
B4GALT2	2.76	0.016
NCOA2	2.72	0.016
CYP2C55	2.61	0.024
CAR5B	2.54	0.001
RBBP5	2.52	0.043
CPSF2	2.41	0.016
AA407270	2.39	0.017
OLFR1412	2.39	0.010
PTPRF	2.38	0.046
2410081M15RIK	2.36	0.045
KNS2	2.35	0.021
LOC100047323	2.33	0.029
GPR3	2.30	0.009
TMED9	2.30	0.049
CFH	2.20	0.038
GDAP1L1	2.17	0.036
MST1	2.14	0.023
1500002O20RIK	2.13	0.008
A930008G19RIK	2.12	0.040
ELF2	2.07	0.002
PLA2G5	1.97	0.025
HRH1	1.97	0.029
HMBS	1.89	0.014
PRH1	1.86	0.047

RDH10	1.85	0.007
RAB13	1.85	0.034
TCHP	1.81	0.016
SLC27A1	1.80	0.037
MRRF	1.79	0.007
SLC4A1	1.76	0.012
OLFR707	1.74	0.008
TFPI	1.71	0.006
OXNAD1	1.71	0.049
DLL1	1.70	0.022
USF2	1.66	0.009
ARID4B	1.62	0.013
TNFAIP8L2	1.60	0.028
METRN	1.59	0.044
DYNLL2	1.59	0.021
PDE1A	1.59	0.013
ZFP101	1.59	0.019
RAB5B	1.58	0.032
SLC12A9	1.57	0.018
CLUAP1	1.57	0.007
LOC669168	1.55	0.039
BICD2	1.53	0.001
ICOS	1.52	0.030
SCYL1	1.51	0.035
FAIM	1.50	0.012
AMY2	1.49	0.025
ZFP60	1.49	0.002
GPN1	1.47	0.004
TRAPPC2	1.47	0.020
SIAT7F	1.46	0.027
PTGS1	1.45	0.048
CLEC4F	1.44	0.042
SLC25A34	1.44	0.030
PDLIM5	1.43	0.019
CASP6	1.42	0.026
HOXB5	1.40	0.008
HIST1H3D	1.39	0.027
AFMID	1.38	0.050
PAIP2B	1.38	0.042
EG545253	1.38	0.009
GSTM6	1.37	0.014
AFMID	1.37	0.038
ZKSCAN14	1.36	0.024
TCF20	1.36	0.018
TMEM19	1.35	0.030
AASS	1.33	0.020
RPL7L1	1.33	0.032

ARMC8	1.32	0.008
HUS1	1.32	0.012
DHDH	1.32	0.009
HERPUD2	1.32	0.033
ZFP326	1.31	0.016
STAB1	1.31	0.031
GPS2	1.30	0.024
MGAT1	1.29	0.011
ELTD1	1.28	0.019
EDNRB	1.28	0.016
LIMK2	1.28	0.049
ABCA3	1.27	0.040
ADSSL1	1.27	0.035
ALG8	1.26	0.033
BAT3	1.26	0.021
D4WSU132E	1.24	0.016
NAGS	1.23	0.030
TCP1	1.23	0.049
SCARF1	1.22	0.012
SERHL	1.22	0.034
DAP3	1.22	0.030
SGPL1	1.21	0.037
2700078K21RIK	1.21	0.017
TRAPPC3	1.21	0.043
TMEM2	1.20	0.036
STAB2	1.20	0.031

Supplementary Table 3. Primer sequences for real-time PCR analysis of mouse gene expression.

Gene Name	Forward Primer	Reverse Primer
Bmal1	CCAAGAAAGTATGGACACAGACAAA	GCATTCTTGATCCTTCCTTGGT
Cidec	ATGGACTACGCCATGAAGTCT	CGGTGCTAACACGACAGGG
Cry1	CTGGCGTGGAAGTCATCGT	CTGTCCGCCATTGAGTTCTATG
Cry2	TGTCCCTTCCTGTGTGGAAGA	GCTCCCAGCTTGGCTTGA
Dbp	CGTGGAGGTGCTTAATGACCTTT	CATGGCCTGGAATGCTTGA
Gapdh	CAAGGTCATCCATGACAACTTTG	GGCCATCCACAGTCTTCTGG
Per1	CCCAGCTTTACCTGCAGAAG	ATGGTCGAAAGGAAGCCTCT
Pgc-1a	TATGGAGTGACATAGAGTGTGCT	CCACTTCAATCCACCCAGAAAG
Nr1d1	CATGGTGCTACTGTGTAAGGTGTGT	CACAGGCGTGCACTCCATAG
Nr1d2	TGAACGCAGGAGGTGTGATTG	GAGGACTGGAAGCTATTCTCAGA
Rorc	TCAGCGCCCTGTGTTTTTC	GAGAACCAGGGCCGTGTAG
Apoa4	CCAGCTAAGCAACAATGCCA	TGGAAGAGGGTACTGAGCTGC
Usf2	GCGTTCGGCGACCACAATA	GACTACGCGGTATGTCACCTG
Ffar3	TTCTGAGCGTGGCCTATCCA	AGACTACACTGACCAGACCAG
Gstm6	ACAGGTCATGGACACTCGAAT	TGGCTTCCGTTTCTCAAAGTC
G6pc	ACTGTGGGCATCAATCTCCTC	CGGGACAGACAGACGTTCAGC
Npas2	CAA CAG ACG GCA GCA TCA TCT	TTC TGA TCC ATG ACA TCC GC
Hnf4a	CACGCGGAGGTCAAGCTAC	CCCAGAGATGGGAGAGGTGAT
Rsk1	CCATCACACACCACGTCAAG	TTGCGTACCAGGAAGACTTTG
